# FAP deficiency attenuates T2DM-associated HFpEF by suppressing the CaMKIIδ-Calcineurin A-NFATc2 signaling pathway

**DOI:** 10.1042/CS20256808

**Published:** 2025-09-02

**Authors:** Chao Li, Xiao Han, Jia-Kang He, Sheng-Xing Tang, Yun-Long Zhang, Xiao-Hong Yu, Lian-Jun Gao

**Affiliations:** 1 Department of Cardiology, First Affiliated Hospital of Dalian Medical University, Dalian, 116011, China; 2 Institute of Cardio-Cerebrovascular Medicine, Central Hospital of Dalian University of Technology, Dalian, 116089, China; 3 Department of Cardiology, Yijishan Hospital of Medical College, Wuhu, 241000, China

**Keywords:** CaMKIIδ-Calcineurin A-NFATc2, Cardiac remodeling, FAP, Heart failure with preserved ejection fraction, Type 2 diabetes mellitus

## Abstract

Heart failure with preserved ejection fraction (HFpEF) represents the initial phase of cardiac dysfunction associated with type 2 diabetes mellitus (T2DM). To date, the pathophysiological mechanisms underlying T2DM-induced HFpEF are complex and elusive. Fibroblast activation protein (FAP) is a prolyl-specific serine protease whose inhibition or vaccination has been shown to enhance cardiac repair following myocardial infarction (MI). However, the role and underlying molecular mechanisms by which abnormal FAP activity promotes the development of T2DM-induced HFpEF remain to be elucidated. In this study, the plasma activity and level of FAP were significantly higher in the T2DM with HFpEF group compared with the healthy control group. Moreover, plasma FAP activity and level were positively correlated with the likelihood of T2DM with HFpEF. To investigate the mechanistic involvement of FAP in the development of T2DM-associated HFpEF, a chronic T2DM mouse model was established. The results revealed that FAP knockout (KO) significantly improved B-type natriuretic peptide (BNP) level and E/A ratios compared with the wildtype (WT) T2DM group. Additionally, FAP KO and FAP inhibitor Talabostat alleviated myocardial inflammation, fibrosis, cardiomyocyte apoptosis, oxidative stress, and energy metabolism dysfunction. Mechanistically, an abnormal increase in FAP triggered the calmodulin-dependent protein kinase δ (CaMKIIδ)-Calcineurin A-NFATc2 signaling pathway, leading to the aforementioned pathological changes in T2DM-induced HFpEF. In contrast, FAP KO suppressed the CaMKIIδ-Calcineurin A-NFATc2 signaling pathway and attenuated these pathological changes. Overall, these findings suggest that FAP may serve as a critical therapeutic target for T2DM-induced HFpEF.

## Introduction

Mounting epidemiological evidence [1] suggests that the global prevalence of diabetes has significantly increased in the past three decades. Between 1990 and 2022, the number of adults diagnosed with diabetes cases surged from 200 million to 828 million worldwide, with China accounting for 148 million cases (18% of the global total), ranking second globally. Diabetes poses a major health threat, especially due to cardiovascular complications, which is responsible for approximately 50–80% of diabetes-related deaths. In 1972, Rubler et al. defined diabetes cardiomyopathy (DCM) as intrinsic myocardial dysfunction [2], which is distinct from atherosclerotic vascular disease. DCM is characterized by structural and functional cardiac abnormalities in the absence of significant hypertension, coronary artery disease (CAD), or valvular disease [3]. It is hallmarked by left ventricular (LV) dilatation, myocardial hypertrophy, myocardial interstitial fibrosis, and diastolic dysfunction. Metabolic alterations in type 2 diabetes mellitus (T2DM) patients predispose them to Heart failure with preserved ejection fraction (HFpEF), which is the predominant type of heart failure (HF) in this population [4,5]. According to the 2023 focused update of the 2021 ESC guidelines for the diagnosis and treatment of acute and chronic heart failure [6,7] and the 2022 AHA/ACC/HFSA guideline for the management of heart failure [8], HFpEF is defined as objective evidence of cardiac structural and/or functional abnormalities consistent with the presence of LV diastolic dysfunction or elevated LV filling pressures, including elevated natriuretic peptide levels despite preserved LV ejection fraction (LVEF ≥ 50%). The etiology of DCM is intricate and not fully understood. Alterations in myocardial cell metabolism are considered key drivers of myocardial dysfunction in diabetes. As is well documented, diabetes can contribute to metabolic abnormalities in myocardial cells, including disruptions in fatty acid and glucose metabolism. In addition, the pathological activation of Ca^2+^/calmodulin-dependent protein kinase δ (CaMKIIδ) [9], a key regulatory protein in the calcium signaling pathway, also plays a pathophysiological role in T2DM-induced HFpEF. These critical mechanisms result in oxidative stress, inflammation, myocardial cell hypertrophy, apoptosis, impaired energy metabolism, and the accumulation of advanced glycation end products (AGEs) and eventually culminate in myocardial dysfunction and HFpEF [9,10]. Notably, traditional glycemic control therapies have demonstrated limited effectiveness in lowering the risk of HF or improving cardiac function. Therefore, there is a pressing need to identify effective treatment strategies for DCM.

Fibroblast activation protein (FAP) is a prolyl-specific serine protease with constitutive activity belonging to the dipeptidyl peptidase-4 (DPP4) family and exists in a soluble circulating form in the bloodstream. It can hydrolyze dipeptidyl and endopeptidase substrates, including naturally occurring bioactive peptides containing denatured collagen and α2-antiplasmin [11]. While generally absent in normal adult tissues, the expression level of FAP increases during tumorigenesis, tissue injury, fibrosis, and inflammation. The role of FAP has been explored in various cardiac diseases, such as post-myocardial infarction (MI) fibrosis and fibrosis induced by angiotensin II administration. According to an earlier study, pharmacological inhibition of FAP enhances B-type natriuretic peptide (BNP) stability, promotes angiogenesis, and restores cardiac function following MI [11]. Indeed, FAP-vaccinated mice demonstrated preserved tissue repair and cardiac structure stability following MI. Besides, cardiac fibrosis was attenuated in FAP-vaccinated mice following angiotensin II injection [12,13]. However, studies investigating the role of FAP in T2DM-associated HFpEF remain scarce. Studies have shown that FAP is a key marker of activated fibroblasts during HFpEF progression. Its overexpression correlates with myocardial fibrosis, diastolic dysfunction, and disease severity [14]. We postulate that aberrant FAP activity may play an instrumental role in the pathogenesis of T2DM-associated HFpEF.

This study aimed to explore the potential effects and molecular mechanisms of FAP in T2DM-associated HFpEF. The results revealed that the plasma activity and level of FAP were significantly higher in the T2DM with HFpEF group compared with the healthy control group. Furthermore, plasma FAP activity and level were positively correlated with the likelihood of T2DM with HFpEF. To examine the mechanistic involvement of FAP in the development of T2DM-associated HFpEF, a chronic T2DM mouse model was generated. The findings showed that FAP knockout (KO) attenuated cardiac hypertrophy, inflammation, oxidative stress, fibrosis, cell apoptosis, and energy metabolism dysfunction. Mechanistically, abnormal FAP activation initiated the CaMKIIδ-Calcineurin A-NFATc2 signaling pathway, which plays a key role in T2DM-induced HFpEF.

## Materials and methods

### Animal Studies

Homozygous FAP KO mice (male, eight weeks old) on a C57BL/6J background and age-matched wildtype (WT) male mice were purchased from GemPharmatech Co., Ltd. (Nanjing, China). All mice are kept in the Animal Center of Dalian Medical University. All mice were maintained on a 12 hour/12 hour light**–**dark cycle with *ad libitum* access to water and food in a specific pathogen-free (SPF) environment with predefined temperature and humidity. Next, the mice were randomly assigned to four groups, namely the WT Sham, FAP KO Sham, WT T2DM, and FAP KO T2DM groups. Mice in the FAP KO T2DM and WT T2DM groups were fed a high-fat diet (60% energy derived from fat, 12492M, Beijing Boaigang Biological Technology Co., Ltd, China) for six weeks, followed by intraperitoneal injection of streptozotocin (STZ) (30 mg/kg body weight, dissolved in citrate buffer at a pH of 4.50, Beijing Boaigang Biological Technology Co., Ltd, China) for several days as described in a previous study [15] and continued to receive the same diet as before for at least eight weeks. Conversely, mice in the WT Sham and FAP KO Sham groups were fed a standard ordinary diet, injected with an equivalent dose of citrate buffer vehicle for the same duration, and continued to be fed a standard diet for the same period. Male WT mice were randomly subjected to Sham or high-fat diet and STZ (HFD + STZ) to make T2DM model and then oral administration with FAP inhibitor (Talabostat, 0.5 mg/kg, one time per day for one week) [16] or vehicle when these mice were fed for 15 weeks. Fasting blood glucose (FBG) levels (tail vein sampling) were monitored weekly using a glucometer (Sinecare, Changsha, China) after fasting for 6–8 hours. FBG levels exceeding 11.2 mmol/l [17] and random blood glucose levels exceeding 16.7 mmol/l [18] were considered indicative of diabetes.

### Glucose and insulin tolerance test

To assess glucose tolerance, an intraperitoneal glucose tolerance test (IPGTT) [14,16] was performed on all mouse groups concurrently on day 7 of Talabostat treatment (24 hours after the final Talabostat administration). To minimize stress responses, all mice underwent a 6-hour daytime fast prior to testing. Glucose (1.5 g/kg body weight) was then administered intraperitoneally. Blood glucose levels were measured immediately before injection (0 minute) and at 30, 60, 90, 120 minutes postinjection.

Insulin sensitivity was evaluated in fasted mice via an intraperitoneal insulin tolerance test (ITT) [14,16]. Insulin (0.75 IU/kg body weight) was injected intraperitoneally. Blood glucose levels were measured preinjection (0 minute) and at 30, 60, 90, 120 minutes postinjection.

### Echocardiography

Eight weeks after achieving diabetic status, transthoracic echocardiographic assessments were performed using a 30-MHz Doppler ultrasound system (SiliconWave, KOLO, Suzhou, China), as outlined in a previous study [19]. The mice were anesthetized using 3.0% isoflurane for induction and then maintained under 1.5% isoflurane. The following structural variables were assessed: average LV anterior wall thickness at diastole and systole (LVAWs, LVAWd), LV posterior wall thickness at diastole and systole (LVPWd, LVPWs), interventricular septal thickness at end-diastole and end-systole (IVSd, IVSs), and LV internal diameters at diastole and systole (LVIDd, LVIDs). LVEF and LV fractional shortening (FS) were calculated as follows: EF = (LV Vol; d - V Vol; s)/LV Vol; d × 100%, FS = (LVIDd - LVIDs)/LVIDd × 100%. To evaluate LV diastolic function, the aortic blood flow in color Doppler mode at the four-chamber view of the heart, the early diastolic blood flow filling (peak E), and the peak atrial systolic blood flow (peak A) were observed and recorded in pulsed Doppler mode. Echocardiographic assessments were conducted in a blinded manner. All mice were euthanized via cervical dislocation under deep anesthesia (2.5% 2,2,2-tribromoethanol, 0.1 ml/10 g body weight), followed by cardiac tissue harvest for subsequent experiments. Drug administration was performed in the designated disposal room, with cardiac tissue collection conducted in the specimen collection room in the Animal Center of Dalian Medical University.

### Western blot analysis

As described in an earlier study [19], total protein samples were extracted from left heart tissues using a lysis buffer containing protease/phosphatase inhibitors (Beyotime, Shanghai, China). Then, the samples were centrifuged, and protein concentrations were quantified using a BCA kit (Thermo Scientific, U.S.A.). Proteins were separated by sodium dodecyl sulfate–polyacrylamide gel electrophoresis (SDS-PAGE) and subsequently transferred to polyvinylidene fluoride membranes (Millipore Corporation, Billerica, MA, U.S.A.). Next, the membranes were blocked with 5% nonfat milk for 1 hour and incubated at 4°C overnight with the primary antibodies for at least 12 hours. Afterward, the membranes were incubated with anti-rabbit IgG or anti-mouse IgG (affinity, Jiangsu, China) secondary antibodies. The Shenhua Technology Chemiluminescence System (Shenhua, Hangzhou, China) was utilized to develop blots, and relative protein levels were quantified using Image J (NIH, Bethesda, Maryland, U.S.A.) with GAPDH serving as an internal control. The primary antibodies used in this study are listed in Supplementary Table–S1.

### Real-time quantitative PCR

Total RNA was extracted from cardiac tissues using TRIzol Reagent (Invitrogen, Carlsbad, CA, U.S.A.) according to the manufacturer’s protocol. Complementary DNA (cDNA) was synthesized by reverse transcription of 1.0 mg RNA using the Evo M-MLV RT Kit with gDNA removal (Accurate Biotechnology, Hunan, China). Quantitative real-time PCR was performed using the SYBR Green Premix Pro Taq HS qPCR Kit (Accurate Biotechnology, Hunan, China) on a Q1000+Real Time qPCR System (LongGene, Hangzhou, China). Relative mRNA expression levels were quantified using the 2^ΔΔCT^ method and normalized to GAPDH. The primer sequences are presented in Supplementary Table–S2.

### Histological analysis

Mouse heart tissues were preserved in 4% paraformaldehyde for at least 48 hours, embedded in paraffin, and sliced into 5 μm-thick transverse sections. Hematoxylin and eosin (H&E), Masson’s trichrome, and wheat germ agglutinin (WGA) staining were carried out using staining kits (H&E and Masson: Servicebio, Beijing, China; WGA: Sigma-Aldrich, St. Louis, MO, U.S.A.). Apoptosis was evaluated using the in situ cell death detection kit (Roche, Basel, Switzerland) following the manufacturer’s protocol. For dihydroethidium (DHE, Sigma-Aldrich, St. Louis, MO, U.S.A.) staining, cardiac sections were incubated with 1 μmol/l DHE in PBS in a humidified box at 37°C for 30 minutes in the dark. For immunofluorescence analysis, cardiac sections were sequentially incubated with a rabbit-derived primary antibody against phosphorylated CaMKIIδ (p-CaMKII), followed by incubation with an Alexa Fluor 555-conjugated anti-rabbit secondary antibody (Beyotime, Shanghai, China). Nuclear counterstaining was performed using DAPI (Beyotime, Shanghai, China). Five random fields of each cardiac sample were captured using a microscope (CX40, SUNNY, Ningbo, China). All images were quantified using ImageJ (V1.53).

### Human studies

All patients provided written informed consent prior to sample collection. The plasma of 30 patients diagnosed with T2DM and comorbid HFpEF based on the 2023 ESC guidelines [20] was collected by qualified cardiologists from the Department of Cardiology between September 2023 and September 2024. Patients with a low ejection fraction (<50%), valvular heart disease, coronary heart disease, dilated or hypertrophic cardiomyopathy, congenital heart disease, hyperthyroidism, inflammatory or systemic diseases, moderate-to-severe renal impairment (estimated glomerular filtration rate <60  ml/min/1.73  m^2^), and malignancies were excluded. Additionally, to mitigate confounding factors linked to HFpEF, patients with hypertension, dyslipidemia, or BMI >35 were excluded. Healthy controls were recruited from the physical examination center and were matched to the experimental group by age and sex. All participants underwent at least one echocardiography examination and blood sample collection. Standardized data collection encompassed laboratory biomarkers (plasma BNP, FBG, glycosylated hemoglobin, uric acid, lipid profile, blood urea nitrogen, and creatinine), demographic characteristics (age, sex, and smoking status (defined as the consumption of >1 cigarette per day for ≥1 year), anthropometric measurements (BMI [weight/height², kg/m²]), morning supine blood pressure using an automated monitor, and echocardiographic indices (LVEF, LVDS, E/A ratio) measured by certified sonographers.

### FAP activity, level, and ATP/AMP assay

FAP levels were quantified in plasma (*n* = 54 per group) and determined using a commercially available colorimetric assay kit (Cloud-Clone Corp, Wuhan, China) according to the manufacturer’s protocol. To assess FAP activity, 1 μl of plasma was diluted in 99 μl of reaction buffer (50 mM Tris-HCl, pH 7.8, 100 mM NaCl), following which 50 μl of this dilution was combined with 50 μM of FAP substrate Suc-Gly-Pro-AMC (MCE, Shanghai, China) in 50 μl of reaction buffer (100 μl total volume). Subsequently, the mixture was transferred to a black 96-well plate (Thermo Scientific, U.S.A.) and incubated at room temperature for 10 minutes. Fluorescence signals were recorded at 5-minute intervals over 0–60 minutes using a fluorimeter set at an excitation wavelength of 380 nm and an emission wavelength of 460 nm. The relative fluorescence unit (RFU) at 60 minutes was subtracted from the RFU at 0 minutes for each well and divided by 60 minutes to determine FAP activity. ATP and AMP concentrations in cardiac tissues were detected using an ELISA kit (Andy gene, Richardson, U.S.A.) according to the protocol.

### Statistical analysis

Continuous variables and categorical variables were presented as mean ± SD and percentages, respectively. Categorical variables were compared using the chi-squared test. Statistical analyses were performed using the Statistical Product and Service Solutions (SPSS) 26.0. The Shapiro**–**Wilk test was employed to evaluate data normality. Differences in normally distributed and non-normally distributed data were assessed using the *t*-test and the Mann**–**Whitney *U* test, respectively. Potential associations between FAP level, FAP activity, and T2DM-induced HFpEF were further explored using multivariable logistic regression analysis, with all models adjusted for age, sex, DBP, LVEF, and LVDS. Receiver operating characteristic (ROC) curves were plotted to assess the sensitivity and specificity of plasma FAP activity and level in predicting T2DM with HFpEF, with the area under the curve (AUC) calculated. A multivariate linear regression model was employed to evaluate the relationship between plasma FAP level, FAP activity, and the E/A ratio. *P*<0.05 was considered statistically significant.

## Results

### Plasma activity and level of FAP were higher in the T2DM with HFpEF group

**Figure 1:**
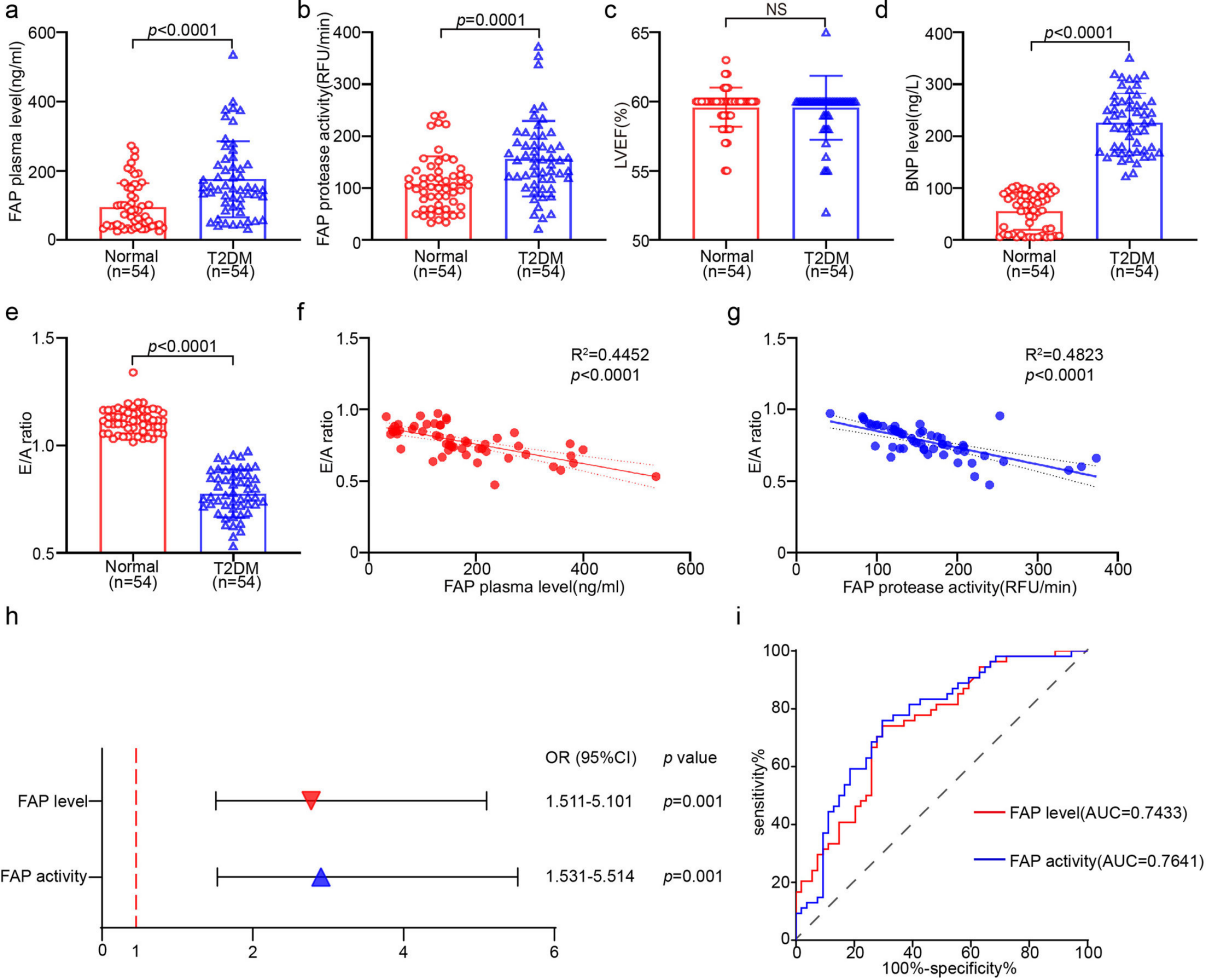
Plasma FAP activity and level elevated in T2DM with HFpEF group. (**a**) Plasma concentration of FAP level by colorimetric kit (*n* = 54 per group); (**b**) Detection of FAP activity in plasma (*n* = 54 per group); (**c**) LVEF obtained by ECHO (*n* = 54 per group); (**d**) BNP level from clinical laboratory (*n* = 54 per group); (**e**) E/A ratio obtained by ECHO (*n* = 54 per group); (**f**) multivariate linear regression analyzed the correlation between the level plasma FAP and E/A ratio; (**g**) multivariate linear regression analyzed the correlation between the actvity plasma FAP and E/A ratio; (**h**) compared with the healthy control group, multivariate logistic regression analysis for the presence of T2DM with HFpEF; (**i**) compared with the healthy control group, receiver operator characteristic curves of plasma FAP activity and level to predict T2DM with HFpEF. Data are expressed as mean ± SD, and n represents the number of samples. FAP, fibroblast activation protein; HFpEF, heart failure with preserved ejection fraction; T2DM, type 2 diabetes mellitus.

The data of 54 patients with T2DM and comorbid HFpEF, as well as 54 healthy controls, were collected. The clinical characteristics of both patient groups are detailed in Table 1. The LVEF in the T2DM with HFpEF group was comparable with the controls, remaining above 50% (Figure 1c and Table 1). However, BNP level and the E/A ratio were significantly higher in the T2DM with HFpEF group compared with the control group (Figure 1d and e). To investigate the relationship between the activity and level of FAP in T2DM with HFpEF patients and controls, the activity and level of FAP were measured. The results unveiled that compared with the control group, plasma FAP activity (164.29 ± 66.67 RFU/min vs. 107.83 ± 53.01 RFU/min) and level (175.60 ± 109.82 ng/ml vs. 95.37 ± 68.17 ng/ml) were significantly higher in the T2DM with HFpEF group (Figure 1a and b). Meanwhile, multivariable logistic regression analysis uncovered a significant positive association between plasma FAP activity (OR 2.906, 95% CI 1.531–5.514; *P*=0.001) and level (OR 2.776, 95% CI 1.511–5.101; *P*=0.001) and the presence of T2DM with HFpEF (Figure 1h and Table 2).

**Table 1:** Clinical and biochemical characteristics of control and T2DM combined with HFpEF patients

Characteristics	The controls (*n* = 54)	T2DM combined with HFpEF (*n* = 54)	*P* value
Clinical parameters			
Age (years)	53.93 ± 9.07	55.28 ± 6.09	0.365
Male, *n* (%)	30 (55.56)	28 (51.85)	0.700
BMI (kg/m^2^)	24.67 ± 3.86	25.03 ± 3.71	0.617
Smoking, *n* (%)	10 (18.52)	8 (14.81)	0.606
SBP (mmHg)	116.37 ± 13.27	123.69 ± 9.45	0.001
DBP (mmHg)	72.83 ± 11.29	78.13 ± 9.18	0.009
Laboratory parameters			
TC (mmol/l)	4.27 ± 0.71	4.08 ± 0.87	0.240
TG (mmol/l)	1.41 ± 1.12	1.33 ± 0.53	0.657
LDL-C (mmol/l)	2.55 ± 0.63	2.35 ± 0.69	0.127
HDL-C (mmol/l)	1.23 ± 0.31	1.07 ± 0.21	0.003
Cr (mmol/l)	61.69 ± 12.57	64.85 ± 15.28	0.243
BUN (mmol/l)	5.57 ± 0.93	5.79 ± 1.32	0.320
UA (mmol/l)	358.35 ± 110.32	330.28 ± 87.57	0.146
FBG (mmol/l)	4.92 ± 0.58	9.24 ± 3.74	<0.001
HbA1c (%)	4.71 ± 0.68	8.03 ± 1.40	<0.001
BNP (ng/l)	55.55 ± 36.13	226.13 ± 56.57	<0.001
Echocardiographic parameters			
LVEF (%)	59.63 ± 1.10	59.56 ± 2.31	0.832
LVDS (mm)	45.54 ± 2.18	46.57 ± 4.30	0.117
E/A ratio	1.12 ± 0.06	0.78 ± 0.11	<0.001
Plasma FAP level (ng/ml)	95.37 ± 68.17	175.60 ± 109.82	<0.001
Plasma FAP activity (RFU/min)	107.83 ± 53.01	164.29 ± 66.67	<0.001

P < 0.05 means statistically significant.

BMI = body mass index. BNP = B-type natriuretic peptide. BUN = blood urea nitrogen. Cr = creatinine. DBP = diastolic blood pressure. FBG = fasting blood glucose. HDL-C = high-density lipoprotein cholesterol. HbA1c = glycosylated hemoglobin (HbA1c values). LDL-C = low-density lipoprotein cholesterol. LVDS = left ventricular end systolic diameter. LVEF = left ventricular ejection fraction. SBP = systolic blood pressure. TC = total cholesterol. TG = triglyceride. UA = uric acid. *P*<0.05, statistically significant. FAP, fibroblast activation protein.

**Table 2:** Multivariable logistic regression model of FAP expression and its activity in patients with T2DM combined with HFpEF

Model	Odds ratios (95% CI)	*P* value
Plasma FAP level (ng/ml)	2.776 (1.511–5.101)	0.001
Plasma FAP activity (RFU/min)	2.906 (1.531–5.514)	0.001

Compared with the control group, all models adjusted for age, gender, DBP, LVEF, and LVDS. *P*<0.05 means statistically significant.

CI, confidence interval. T2DM, type 2 diabetes mellitus. FAP, fibroblast activation protein. HFpEF, heart failure with preserved ejection fraction. LVDS, left ventricular end-systolic diameter. LVEF, left ventricular ejection fraction.

ROC analysis was performed to determine the predictive value of plasma FAP activity and level for T2DM with HFpEF. The AUC for plasma FAP level for predicting T2DM with HFpEF was 0.7433 (95% CI: 0.6511–0.8355, *P*<0.0001). The optimal cutoff value for plasma FAP level on the ROC curve was 107.22 μmol/l, yielding a sensitivity of 74.1% and specificity of 70.4%. Regarding plasma FAP activity, the AUC was 0.7641 (95% CI: 0.6735–0.8546, *P<*0.0001), and the optimal cutoff value was 121.01 RFU/min, with a sensitivity of 75.9% and specificity of 70.4% (Figure 1i).

Multivariate linear regression models were utilized to assess the relationship between plasma FAP level, FAP activity, and the E/A ratio in T2DM with HFpEF. As anticipated, plasma FAP level (*R*² = 0.4452; *P<*0.0001) and FAP activity (*R*² = 0.4823; *P*<0.0001) were strongly associated with the E/A ratio obtained from cardiac echocardiography. More importantly, the correlation remained significant after adjusting for age, sex, triglyceride levels, diastolic blood pressure (DBP), LVEF, and left ventricular end-systolic diameter (LVDS) in all models (Figure 1f and g).

### FAP KO significantly decreased FAP activity and level in T2DM-induced HFpEF mice without altering blood glucose levels or body weight

**Figure 2:**
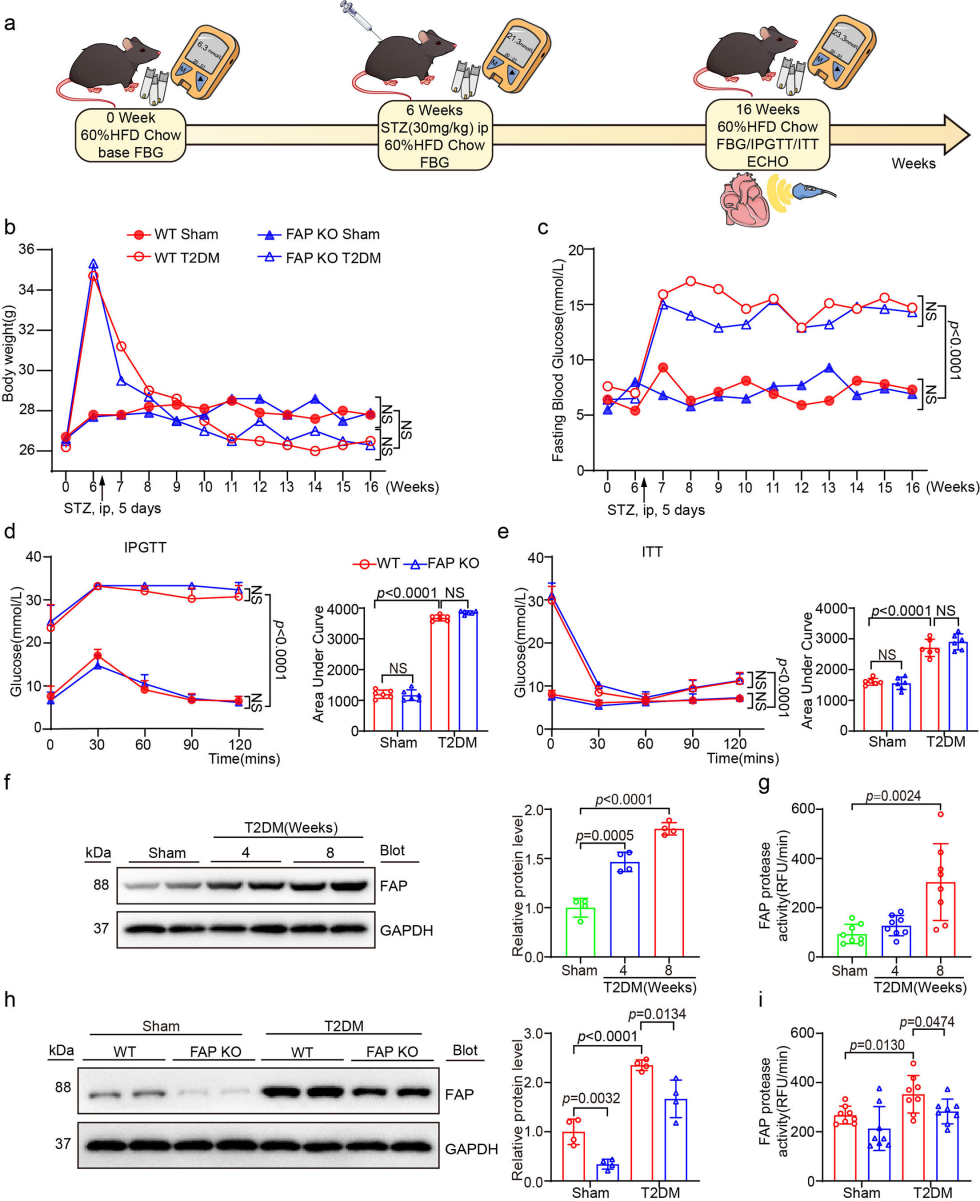
FAP activity and level are increased in the heart of mice from the T2DM-induced HFpEF group. After FAP KO, FAP activity and level are decreased. (**a**) Experimental animal operation and medication flow chart; (**b**) average body weight of the mice in each group (*n* = 8 per group); (**c**) average fast blood glucose of the mice in each group (*n* = 8 per group); (**d**) IPGTT with WT or FAP KO mice in Sham, T2DM group and AUC analysis; (**e**) ITT with WT or FAP KO mice in Sham, T2DM group and AUC analysis; (**f**) FAP were analyzed by western blotting for protein level in continuing high-fat diet for four and eight weeks after T2DM induction by STZ intraperitoneal injection (left). Quantification of the relative protein level (right, *n* = 4 per group); (**g**) FAP activity in heart of mice from high-fat diet for four and eight weeks after T2DM induction by STZ intraperitoneal injection (*n* = 8 per group); (**H**) FAP protein level in heart of mice from continuing high-fat diet for eight weeks after T2DM induction by STZ intraperitoneal injection with WT or FAP KO mice in Sham, T2DM group (left) and quantification of the relative protein level (right, *n* = 4 per group); (**i**) FAP activity in heart of mice from high-fat diet for eight weeks after T2DM induction by STZ intraperitoneal injection with WT or FAP KO mice in Sham, T2DM group (*n* = 8 per group). Data are expressed as mean ± SD, and *n* represents the number of samples. FAP, fibroblast activation protein; HFpEF, heart failure with preserved ejection fraction; KO, knockout; STZ, streptozotocin; T2DM, type 2 diabetes mellitus.

Based on the aforementioned findings regarding FAP activity and level in human plasma, a T2DM model was constructed in mice through a HFD + STZ administration to induce HFpEF (Figure 2a). FBG levels were over 11.2 mmol/l, while random blood glucose levels exceeded 16.7 mmol/l in both the T2DM-induced HFpEF FAP KO group and WT group (Figure 2c). At the same time, there was a significant statistical difference in IPGTT and ITT between the T2DM group and the Sham group. The results demonstrated significant glucose intolerance and insulin resistance in T2DM mice compared with the Sham group (Figure 2d and e). However, no significant difference in body weight was observed between the T2DM-induced HFpEF and control groups (Figure 2b). In order to assess FAP expression in the cardiac tissue of T2DM-induced HFpEF mice, FAP activity and protein level were quantified after four and eight weeks of continued high-fat diet and T2DM induction via intraperitoneal STZ injection. After the successful construction of the T2DM model and continuous feeding of an HFD for eight weeks, both FAP activity and level remained significantly elevated (Figure 2f and g). Therefore, the model involving eight weeks of continued HFD feeding followed by HFD + STZ induction was selected to construct the T2DM-induced HFpEF model in mice, which was used for the ensuing experiments (Figure 2a).

At the same time, FAP activity and level were assessed in FAP KO and WT mice under both normal dietary conditions and in the T2DM-induced HFpEF model. The results indicated that in the WT T2DM-induced HFpEF group, FAP activity and level were significantly elevated, whereas these increases were significantly attenuated in FAP KO mice (Figure 2h and i). At the same time, no significant differences in blood glucose levels, weight, IPGTT, and ITT were noted between the FAP KO and WT T2DM-induced HFpEF groups, indicating that FAP KO decreased FAP activity and level without altering blood glucose levels, weight, IPGTT, and ITT in T2DM-induced HFpEF mice (Figure 2b, c, d and e).

### FAP deficiency improved cardiac dysfunction and remodeling in T2DM-induced HFpEF mice

**Figure 3:**
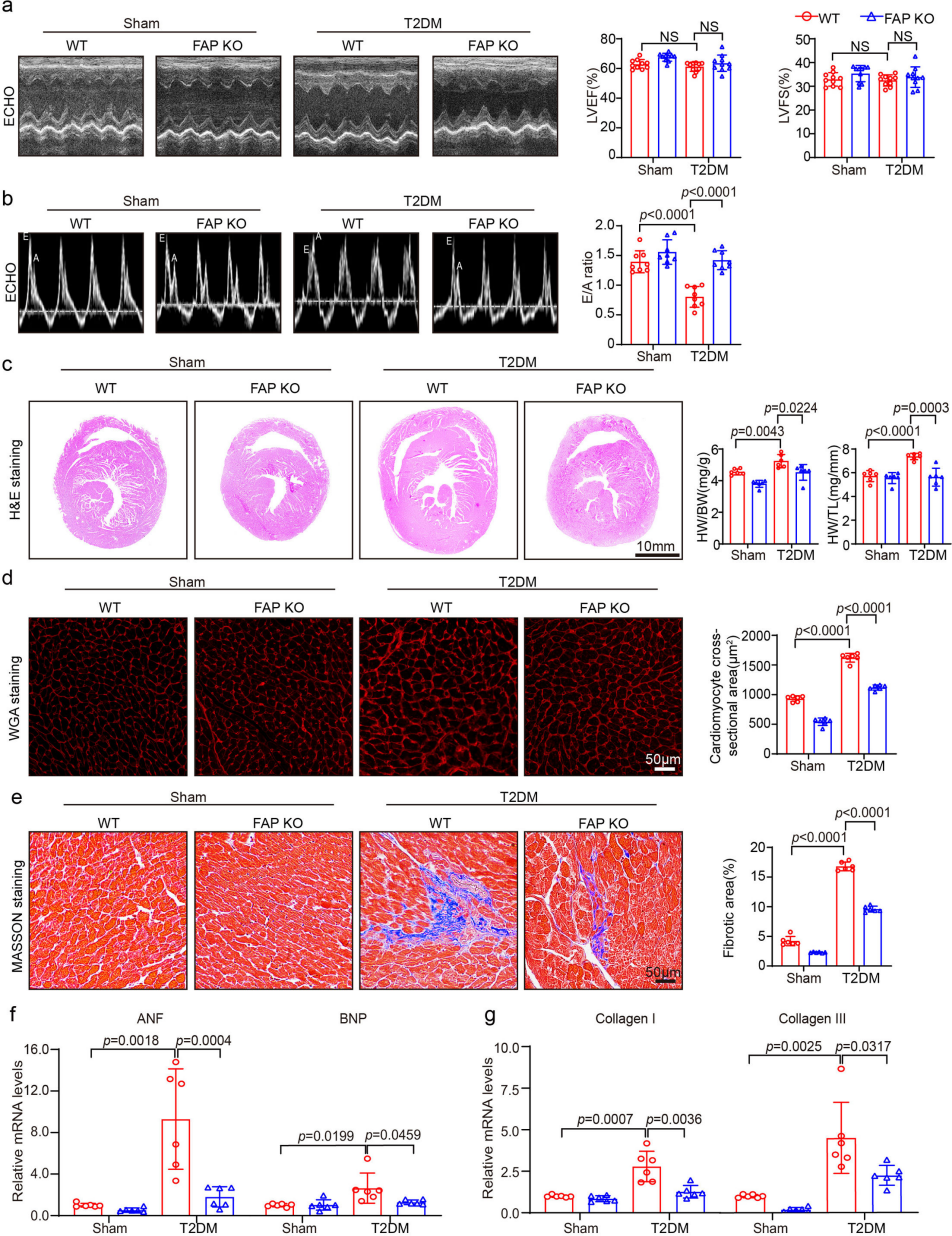
FAP deficiency improved cardiac dysfunction and remodeling in T2DM-induced HFpEF mice. (**a**) M-mode echocardiography of LV chamber (left), measurements of EF%, and FS% in mice (right, *n* = 10 per group); (**b**) transmitral E/A ratio by ECHO (*n* = 8 per group); (**c**) FAP KO mice and WT mice were fed with high-fat diet (60% energy from fat) for six weeks, injected with 30 mg/kg STZ solution intraperitoneally for several days and continued to be fed with same diet as before for at least eight weeks. Representative images of heart sections (left, *n* = 6 per group). Scale bar: 10 mm. The ratios of heart weight to body weight (HW/BW) and heart weight tibial length (HW/TL) (right, *n* = 6 per group); (**d**) assessment of cardiomyocyte hypertrophy: representative micrographs of TRITC-conjugated wheat germ agglutinin (WGA) staining delineating cardiomyocyte boundaries (left). Scale bar: 50 μm. Quantitative analysis of myocyte cross-sectional area (right, *n* = 6 per group, biological replicates with 150–200 cells analyzed per sample); (**e**) evaluation of myocardial fibrosis: characteristic Masson’s trichrome staining showing collagen deposition in myocardial sections (left). Scale bar: 50 μm. Quantification of fibrotic area (%) (right, *n* = 6 per group); (**f**) qPCR analyses of ANF and BNP mRNA levels in the heart (*n* = 6 per group); (**g**) qPCR analyses of Collagen I and Collagen III mRNA levels (*n* = 6 per group). Data are expressed as mean ± SD, and *n* represents the number of samples. FAP, fibroblast activation protein; HFpEF, heart failure with preserved ejection fraction; KO, knockout; STZ, streptozotocin; T2DM, type 2 diabetes mellitus.

To investigate the effect of FAP KO on cardiac function in diabetic mice, echocardiography was carried out to measure cardiac parameters. After eight weeks of diabetes induction, LVEF and LVFS were not significantly decreased compared with the control group (Figure 3a and Table 3). However, the E/A ratio, an indicator reflecting HFpEF [21], was significantly decreased in the WT T2DM-induced HFpEF group and improved following FAP KO in the T2DM-induced HFpEF model (Figure 3b), signaling that FAP KO assisted in mitigating cardiac dysfunction in mice with T2DM-induced HFpEF. A previous study [11] established that BNP is a substrate for FAP. Consequently, the expression level of BNP was examined. The protein level of BNP was significantly up-regulated in the WT T2DM-induced HFpEF group, whereas FAP KO reduced BNP expression level (Supplementary Figure–S1A). Collectively, these results imply that FAP deficiency confers protective effects against HFpEF development in diabetic hearts.

**Table 3:** Cardiac echocardiographic parameters in WT and FAP KO mice with different types of diet

Characteristics	WT Sham	FAP KO Sham	WT T2DM	FAP KO T2DM
LVEF	62.86 ± 3.15	67.58 ± 2.66	61.15 ± 3.10	63.52 ± 5.50
LVFS	33.32 ± 2.36	36.98 ± 2.15	32.12 ± 1.97	33.87 ± 4.32
E/A	1.39 ± 0.18	1.56 ± 0.21	0.80 ± 0.17^a^	1.42 ± 0.16 *b*
LVAW;d	0.78 ± 0.19	0.90 ± 0.13	0.85 ± 0.13	0.86 ± 0.17
LVAW;s	2.37 ± 1.34	2.10 ± 1.23	2.12 ± 1.13	2.86 ± 1.18
LVID;d	2.09 ± 1.45	2.80 ± 1.43	2.47 ± 1.43	1.69 ± 1.31
LVID;s	1.86 ± 0.50	2.15 ± 0.34	1.97 ± 0.55	1.70 ± 0.48
LVPW;d	1.61 ± 0.88	1.31 ± 0.72	1.48 ± 0.85	1.92 ± 0.82
LVPW;s	1.02 ± 0.16	1.22 ± 0.12	1.09 ± 0.11	1.15 ± 0.12

a *P*<0.05 versus WT Sham.

b *P*<0.05 versus WT T2DM.

LVPW: left ventricular posterior wall.

Histological evaluations were performed across groups to determine the effects of FAP KO on myocardial hypertrophy and cardiac fibrosis. Compared with controls, extensive myocardial hypertrophy and cardiac fibrosis were detected in the WT T2DM-induced HFpEF group, as evidenced by higher heart weight-to-body (HW/BW) and heart weight-to-tibial length ratios (HW/TL), as well as higher LV wall thickness. However, FAP KO reversed these changes (Figure 3c). Next, WGA staining was performed to evaluate the cross-sectional area of cardiomyocytes, and the results suggested that compared with the non-diabetic control group, hearts from the WT T2DM-induced HFpEF group were larger and displayed signs of sustained hypertrophy. However, FAP KO alleviated these changes (Figure 3d). In addition, the levels of ANF and BNP were significantly higher in the WT T2DM-induced HFpEF group. These abnormalities were significantly alleviated after FAP KO (Figure 3f). In addition, compared with the non-T2DM group, WT T2DM-induced HFpEF mice subjected to eight weeks of hyperglycemia exhibited extensive LV fibrosis, manifested by increased fibrosis areas and higher mRNA expression levels of fibrosis markers (Collagens I and III) (Figure 3e and g). After FAP KO, the aforementioned changes were attenuated compared with the WT T2DM-induced HFpEF group.

### FAP KO substantially alleviated inflammation and oxidative stress in T2DM-induced HFpEF

**Figure 4:**
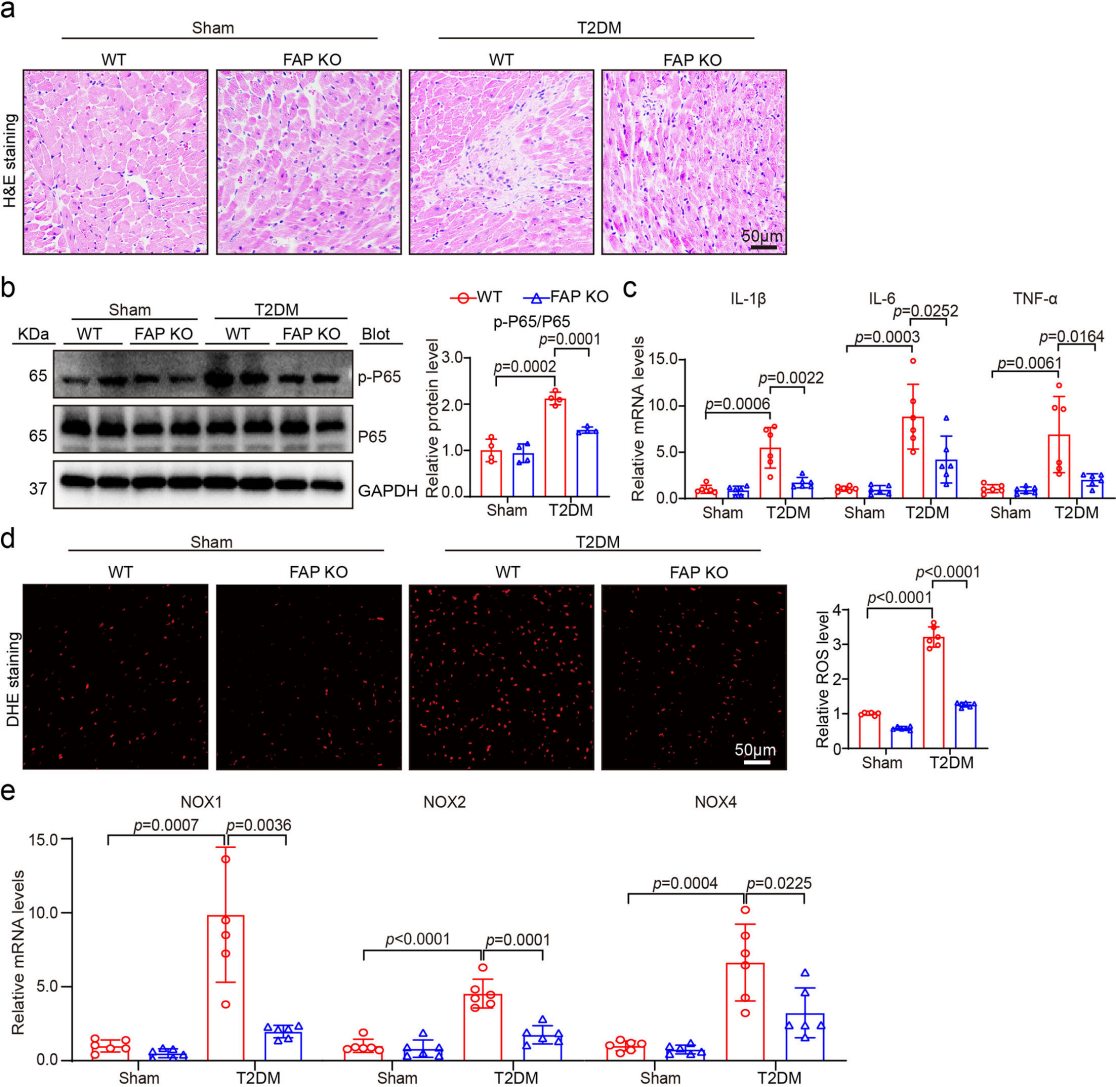
FAP KO remarkably reduced inflammation and oxidative stress in T2DM-induced HFpEF. (**a**) FAP KO mice and WT mice were fed with high-fat diet (60% energy from fat) for six weeks, injected with 30 mg/kg STZ solution intraperitoneally for several days and continued to be fed with same diet as before for at least eight weeks. Representative images of H&E staining of heart sections. Scale bar: 50 μm; (**b**) immunoblotting analyses of p-P65, P65, and GAPDH in the heart (left). Quantification of the relative protein levels (right, *n* = 4 per group); (**c**) qPCR analyses of IL-1β, IL-6, and TNF-α mRNA levels (*n* = 6 per group); (**d**) representative images of dihydroethidium (DHE) staining of the heart sections (left). Quantification of the relative superoxide production (right, *n* = 6 per group). Scale bar: 50 μm; (**e**) qPCR analyses of NOX1, NOX2 and NOX4 mRNA levels (*n* = 6 per group). Data are expressed as mean ± SD, and *n* represents the number of samples. FAP, fibroblast activation protein; HFpEF, heart failure with preserved ejection fraction; KO, knockout; T2DM, type 2 diabetes mellitus.

To evaluate the effects of FAP KO on inflammation and superoxide production generation, additional investigations were conducted to assess inflammation and oxidative stress in cardiac tissue from T2DM-induced HFpEF models. Sustaining eight weeks of high-glucose infiltration enhanced LV inflammatory responses compared with non-T2DM controls. At the same time, the levels of inflammatory cell infiltration, proinflammatory cytokines (IL-1 β, IL-6, and TNF-α), and phosphorylation expression of p65 were increased (Figure 4a, b and c). Following FAP KO, these pathological manifestations were significantly attenuated compared with the WT T2DM-induced HFpEF group. Moreover, DHE staining displayed that T2DM-induced HFpEF in WT hearts significantly increased DHE intensity (Figure 4d). Finally, the RNA expression levels of oxidative stress markers (NOX1, NOX2, and NOX4) were markedly higher in the WT T2DM-induced HFpEF group, while these elevations were substantially decreased following FAP KO (Figure 4e).

### FAP KO alleviated T2DM-induced HFpEF-associated apoptosis and energy metabolism dysfunction in mice

**Figure 5:**
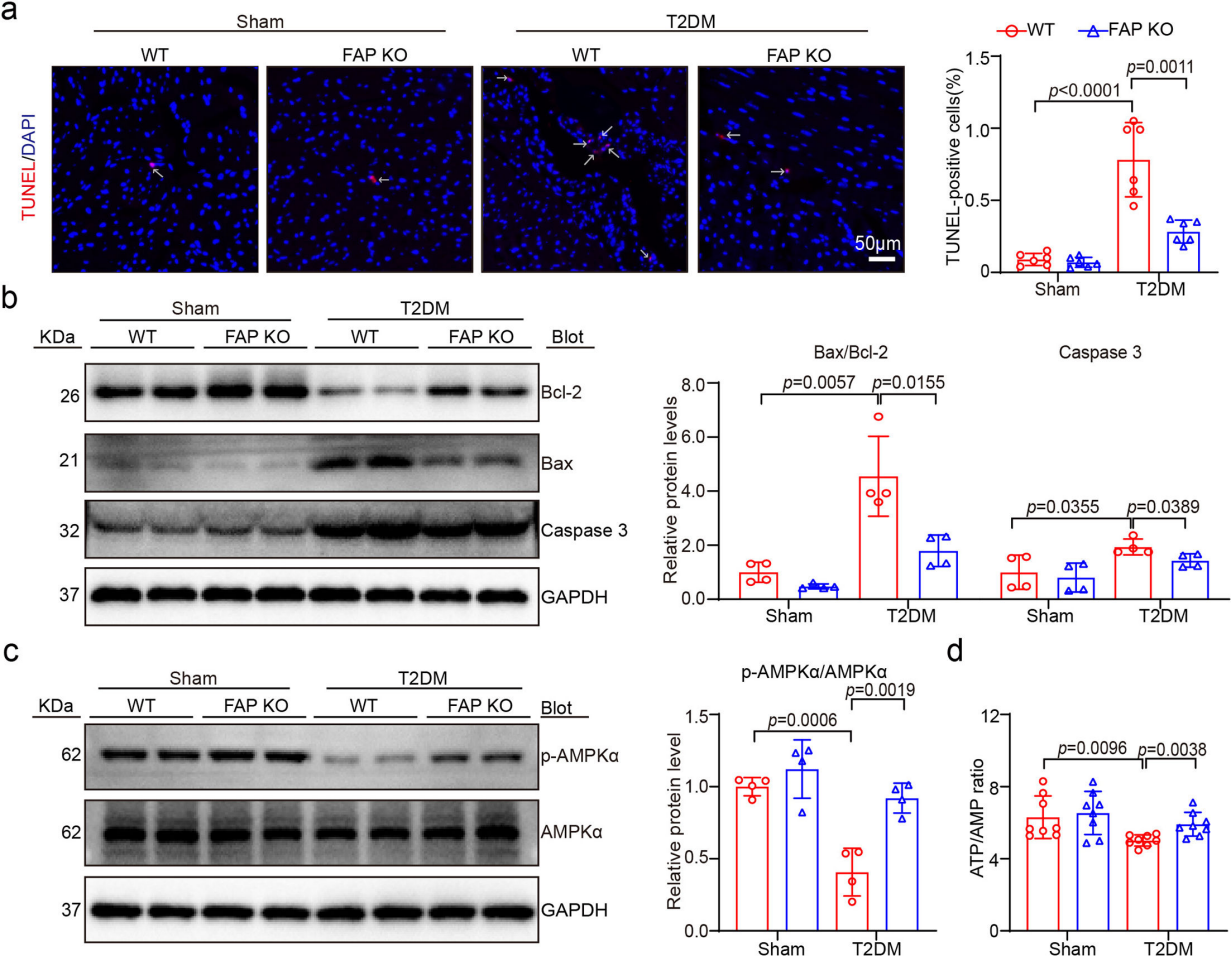
FAP KO alleviated T2DM-induced HFpEF, inducing apoptosis and energy metabolic dysfunction in mice. (**a**) Representative images of TUNEL (red) and DAPI (blue) staining of the heart sections (left), and quantification of TUNEL-positive nuclei (right, *n* = 6 per group). Scale bar: 50 μm; (**b**) immunoblotting analyses of Caspase-3, Bcl-2, Bax, and GAPDH in the heart (left). Quantification of the relative protein levels (right, *n* = 4 per group); (**c**) immunoblotting analyses of p-AMPKα, AMPKα, and GAPDH in the heart (left). Quantification of the relative protein levels (right, *n* = 4 per group); (**d**) AMP in heart of mice from high-fat diet for eight weeks after T2DM induction by STZ intraperitoneal injection by colorimetric kit (left, *n* = 8 per group), ATP in heart of mice by colorimetric kit (middle, *n* = 8 per group) and ATP/AMP ratio (right, *n* = 8 per group). Data are expressed as mean ± SD, and *n* represents the number of samples. FAP, fibroblast activation protein; HFpEF, heart failure with preserved ejection fraction; KO, knockout; T2DM, type 2 diabetes mellitus.

Given that T2DM frequently drives high-glucose infiltration into myocardial cells [9], leading to exacerbated apoptosis, energy metabolism dysfunction, and HFpEF [22], the effects of FAP KO on cell apoptosis and energy metabolism dysfunction, major causes of myocardial dysfunction, were investigated. TUNEL assays exposed that in the WT T2DM-induced HFpEF group, the proportion of TUNEL-positive cells was significantly higher compared with the normal diet control group. However, FAP KO significantly improved these effects. Additionally, the protein expression levels of apoptosis-regulating proteins, such as the Bax/Bcl-2 ratio and Caspase-3, were significantly increased (Figure 5a and b). In contrast, the expression level of p-AMPKα was significantly lower in the WT T2DM-induced HFpEF group compared with the control group. Notably, FAP KO markedly improved energy metabolism and concurrently up-regulated p-AMPKα expression (Figure 5c). Although there was no statistically significant difference in ATP level between the groups, a significant difference was observed in the ATP/AMP ratio, signifying that ATP production was significantly inhibited, and these changes were markedly improved following FAP KO (Figure 5d).

### FAP regulated the pathophysiological response of T2DM-induced HFpEF by activating the CaMKIIδ-Calcineurin A-NFATc2 signaling pathway in mice

**Figure 6:**
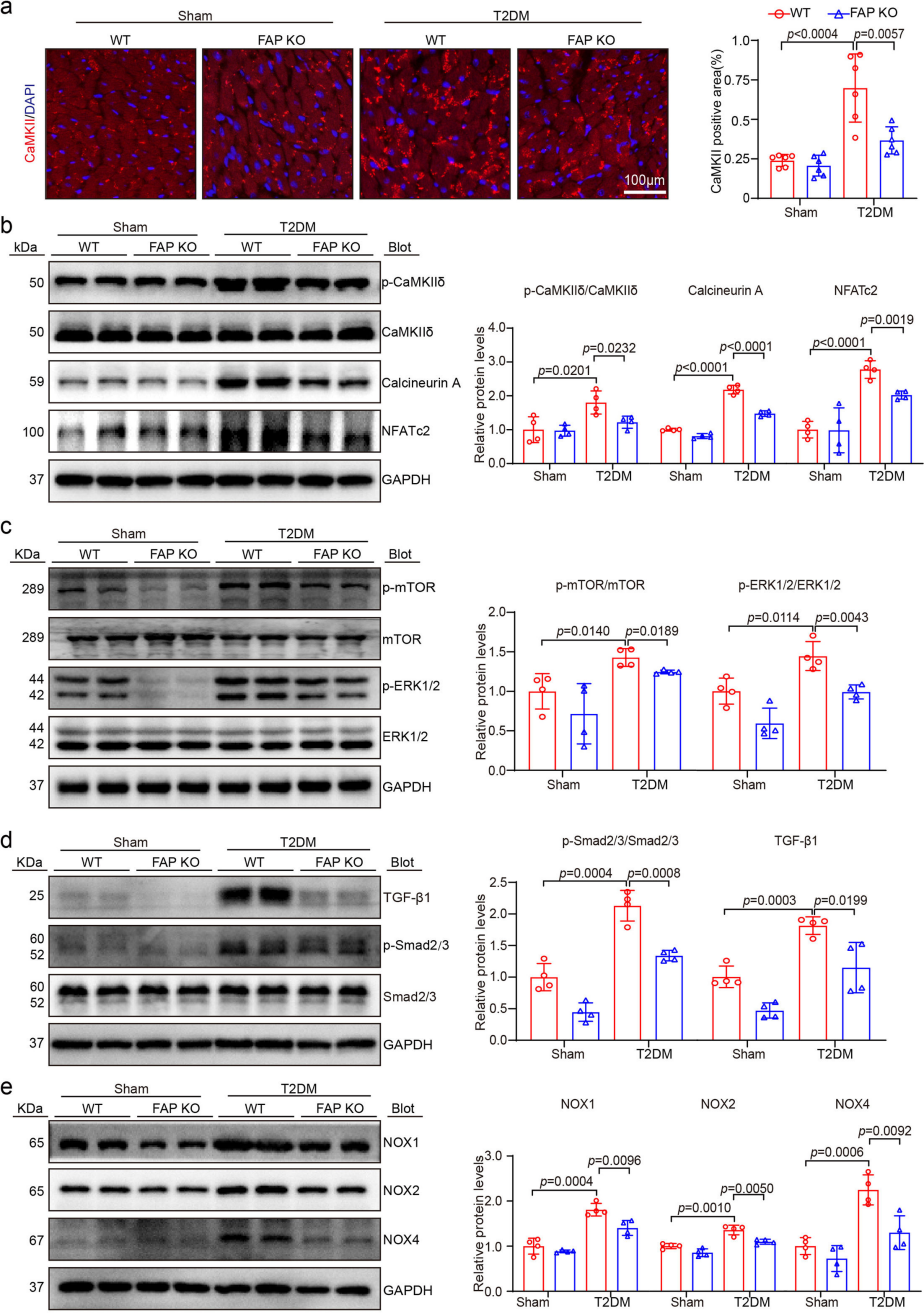
FAP deficiency disrupted the transduction of multiple signaling pathways. (**a**) CaMKII (phosphorylation) immunofluorescent staining (red) and DAPI (blue) staining of the heart sections (left), and quantification of CaMKII (phosphorylation) positive area (right, *n* = 6 per group). Scale bar: 100 μm; (**b**) representative immunoblotting of p-CaMKIIδ, Calcineurin A, NFATc2, and GAPDH in the heart (left). Quantification of the relative protein levels (right, *n* = 4 per group); (**c**) representative immunoblots of p-mTOR, mTOR, p-ERK1/2 and ERK1/2, and GAPDH in the heart (left). Quantification of the relative protein levels (right, *n* = 4 per group); (**d**) representative immunoblots of TGF-β1, p-Smad2/3, Smad2/3 and GAPDH in the heart (left). Quantification of the relative protein levels (right, *n* = 4 per group); (**e**) representative immunoblots of NOX1, NOX2, NOX4, and GAPDH in the heart (left). Quantification of the relative protein levels (right, *n* = 4 per group). Data are expressed as mean ± SD, and *n* represents the number of samples.

Given that the role of FAP in T2DM-induced HFpEF is unclear, the signaling pathways implicated in myocardial hypertrophy, fibrosis, inflammation, oxidative stress, apoptosis, and energy metabolism dysfunction such as the p-mTOR, p-ERK1/2, TGF-β1, p-Smad2/3, NOX1, NOX2, and NOX4 were explored. Interestingly, the expression levels of these proteins were up-regulated in the T2DM-induced HFpEF group. However, their expression levels were down-regulated following FAP KO, suggesting that myocardial hypertrophy, fibrosis, inflammation, oxidative stress, cell apoptosis, and energy metabolism dysfunction were attenuated following FAP KO in the T2DM-induced HFpEF group (Figure 6c, d and e). Moreover, accumulating evidence suggests that BNP acts as an essential substrate for FAP and is involved in the pathogenesis of T2DM-induced HFpEF [11]. Importantly, earlier studies reported that BNP, serving as a critical upstream modulator of CaMK, may play a pivotal role in the abnormal activation of the calcium signaling pathway, which represents a fundamental pathophysiological characteristic of T2DM-induced HFpEF [23,24]. Consequently, the effect of FAP on the CaMKIIδ-Calcineurin A-NFATc2 signaling pathway was examined in T2DM-induced HFpEF. Compared with the control group, the protein levels of p-CaMKIIδ, Calcineurin A, and NFATc2 were significantly up-regulated in WT mice with T2DM-induced HFpEF. Moreover, FAO KO resulted in a marked reduction in the expression levels of p-CaMKIIδ, Calcineurin A, and NFATc2, whereas total CaMKIIδ expression remained unaltered across all experimental groups. These results conjointly position FAP as an upstream regulator of the CaMKIIδ-Calcineurin A-NFATc2 signaling pathway in T2DM-induced HFpEF (Figure 6a and b). Finally, FAP KO negatively regulated the CaMKIIδ-Calcineurin A-NFATc2 signaling pathway, thereby alleviating myocardial hypertrophy, fibrosis, inflammation, oxidative stress, apoptosis, and energy metabolism dysfunction in T2DM-induced HFpEF mice.

### Talabostat, a FAP inhibitor, attenuated cardiac hypertrophy, oxidative stress, and remodeling in T2DM-related HFpEF mice

**Figure 7:**
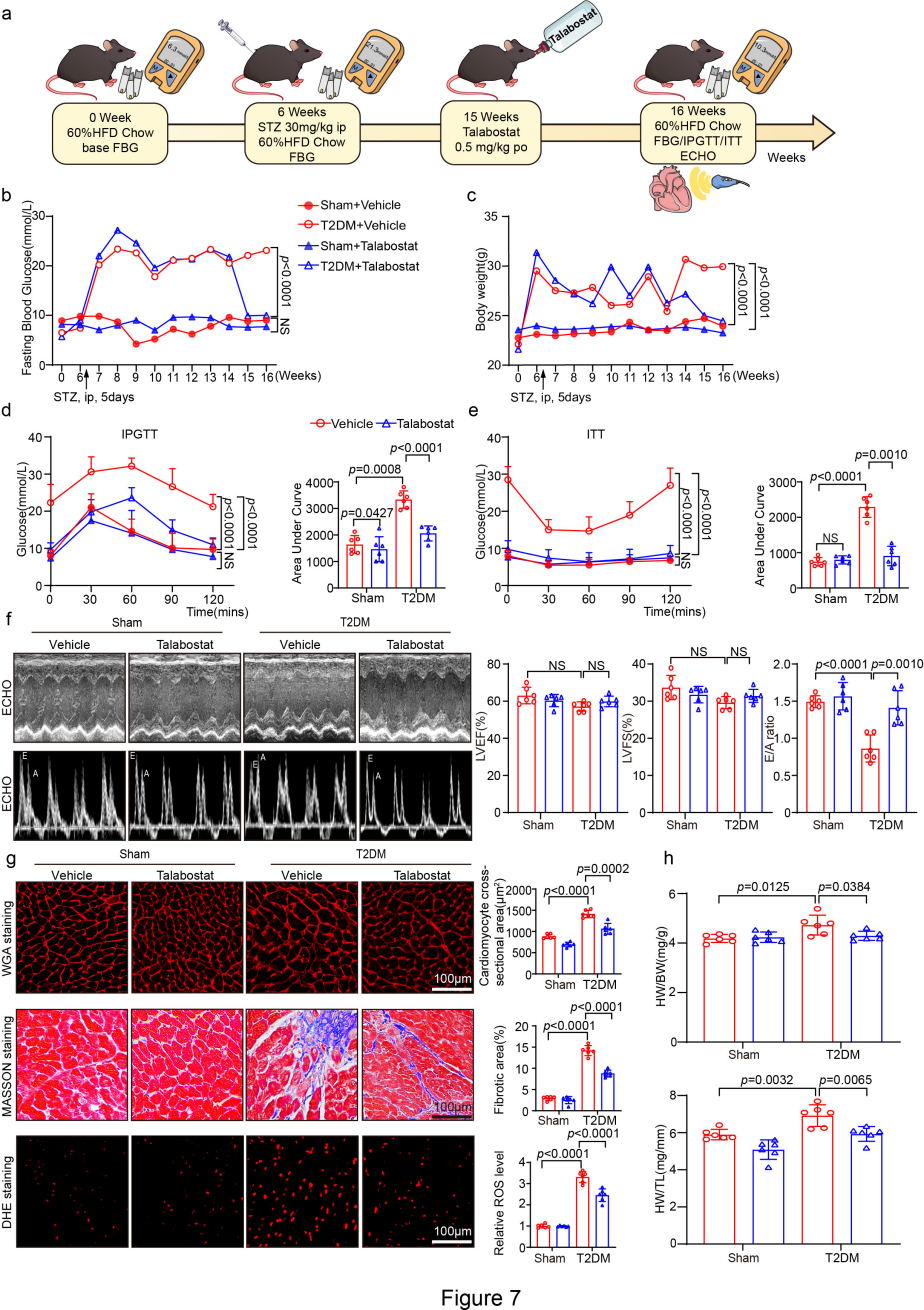
FAP inhibitor Talabostat effectively mitigated multiple pathological processes including cardiomyocyte hypertrophy, myocardial fibrosis, oxidative damage, and metabolic dysregulation, ultimately preserving cardiac structure and function in T2DM-induced HFpEF. (**a**) Experimental animal operation and medication flow chart; (**b**) average fast blood glucose of the mice in each group (*n* = 8); (**c**) average body weight of the mice in each group (*n* = 8); (**d**) IPGTT with Vehicle or Talabostat mice in Sham, T2DM group and AUC analysis; (**e**) ITT with Vehicle or Talabostat mice in Sham, T2DM group and AUC analysis; (**f**) M-mode echocardiography of LV chamber (left) measurements of EF%, FS% in mice (right, *n* = 6 per group) and transmitral E/A ratio by ECHO (*n* = 6 per group); (**g**) assessment of cardiomyocyte hypertrophy: representative micrographs of TRITC-conjugated wheat germ agglutinin (WGA) staining delineating cardiomyocyte boundaries (left). Scale bar : 100 μm. Quantitative analysis of myocyte cross-sectional area (right, *n* = 6 per group, biological replicates with 150–200 cells analyzed per sample); evaluation of myocardial fibrosis: characteristic Masson’s trichrome staining showing collagen deposition in myocardial sections (left). Scale bar: 100 μm. Quantification of fibrotic area (%) (right, *n* = 6 per group); representative images of DHE staining of the heart sections (left). Quantification of the relative superoxide production (right, *n* = 6 per group). Scale bar: 100 μm; (**h**) The ratios of heart weight to body weight (HW/BW) and heart weight tibial length (HW/TL) (right, *n* = 6 per group); Data are expressed as mean ± SD, and *n* represents the number of samples. AUC, area under the curve; DHE, dihydroethidium; FAP, fibroblast activation protein; HFpEF, heart failure with preserved ejection fraction; T2DM, type 2 diabetes mellitus.

To further evaluate whether inhibiting FAP can reverse T2DM-induced HFpEF, we extensively screened FAP inhibitors that can be used for clinical translation and found that Talabostat inhibited activity and level of FAP, which has been used in phase I clinical trials [16]. Next, we evaluated the Talabostat inhibitory effect on activity and level of FAP as well as its action on T2DM-induced HFpEF treatment in mice. WT mice were used to construct a T2DM model by HFD + STZ. After seven weeks of T2DM, they were randomly assigned to give only vehicle or Talabostat for another week (Figure 7a). Compared with T2DM-induced HFpEF mice for eight weeks, the weight and blood glucose levels of T2DM-induced HFpEF mice treated with Talabostat were significantly declined (Figure 7b and c), and the peak blood glucose levels detected by IPGTT and ITT were also significantly different from those of the T2DM-induced HFpEF group (Figure 7d and e). Moreover, significant reversal of cardiac diastolic dysfunction, hypertrophy, myocardial fibrosis, and ROS was observed in T2DM-induced HFpEF mice treated with Talabostat (Figure 7f, g and h), indicating that Talabostat reversed compensatory hypertrophy, cardiac remodeling, and cardiac diastolic dysfunction. In the Sham group, no significant differences were observed in these variables between Talabostat and vehicle groups. In addition, in the HFpEF or Sham group induced by T2DM, the level and activity of FAP decreased in mice treated with Talabostat (Supplementary Figure–S1b. c). These results indicate that FAP is a potential therapeutic target for treating T2DM-induced HFpEF.

## Discussion

**Figure 8:**
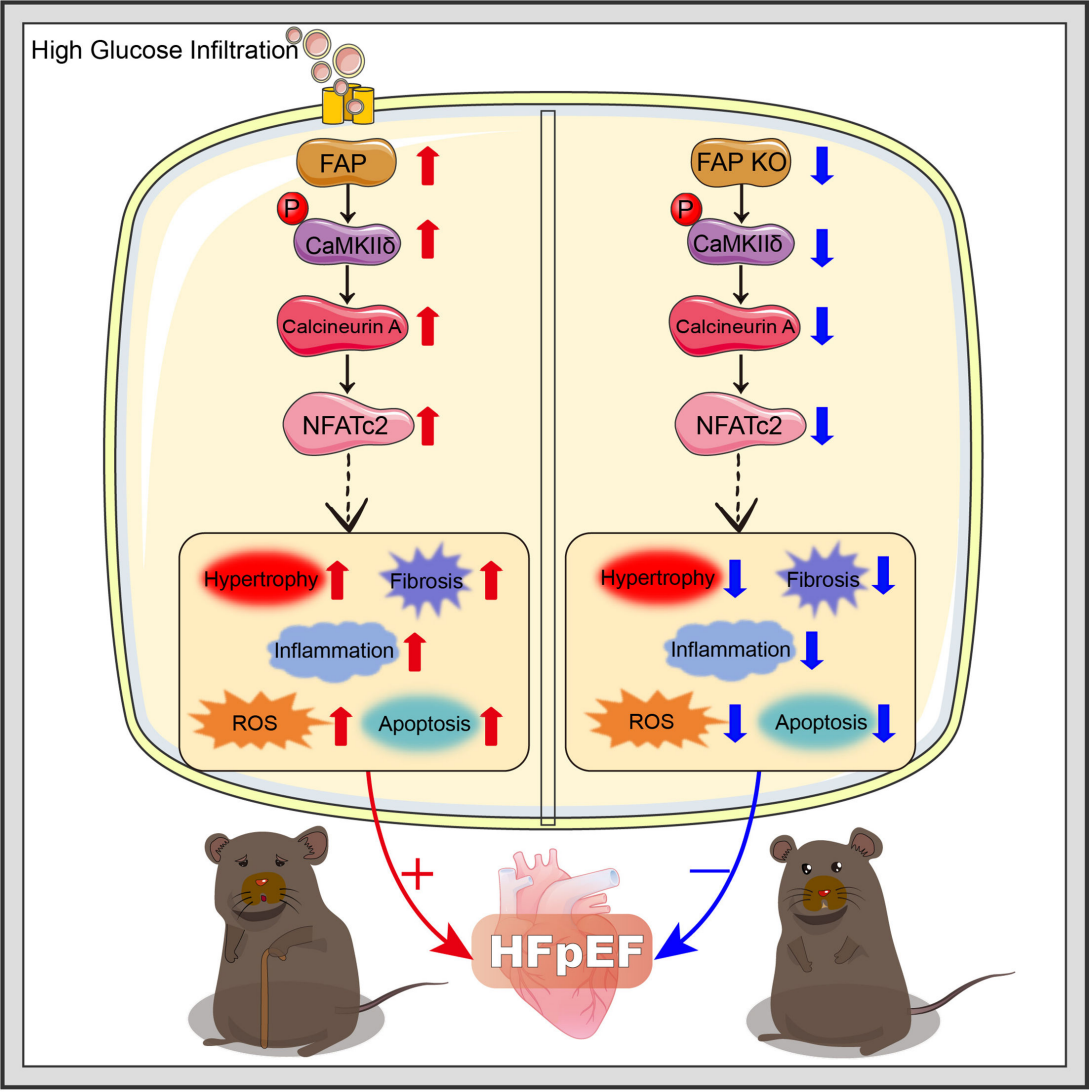
A working model demonstrated that FAP KO significantly attenuated T2DM-induced HFpEF through inhibition of the CaMKIIδ-Calcineurin A-NFATc2 signaling pathway. This effect effectively mitigated multiple pathological processes including cardiomyocyte hypertrophy, myocardial fibrosis, inflammatory infiltration, oxidative damage, cellular apoptosis, and metabolic dysregulation, ultimately preserving cardiac structure and function in T2DM-induced HFpEF. FAP, fibroblast activation protein; HFpEF, heart failure with preserved ejection fraction; KO, knockout; T2DM, type 2 diabetes mellitus.

DCM is a unique and severe cardiac complication that occurs independently of other diabetes-related complications. Prolonged chronic hyperglycemia progressively impairs cardiac structure and function, initially manifested as structural remodeling [22]. The precise pathophysiological role of FAP in the progression of T2DM-associated HFpEF remains a critical knowledge gap. Emerging evidence suggests FAP may synergistically interact with diabetic metabolic derangements (hyperglycemia and insulin resistance) to exacerbate myocardial stiffness, which is a hallmark of HFpEF [25]. This study demonstrated that FAP plays a decisive role in T2DM-associated HFpEF by activating the CaMKIIδ-Calcineurin A-NFATc2 signaling pathway, ultimately promoting myocardial hypertrophy, fibrosis, inflammation, oxidative stress, apoptosis, and energy metabolism dysfunction (Figure 8). DCM is characterized by a progression from early molecular changes to mid-stage structural changes and significant impairment of diastolic function. Our findings suggested that BNP level and the E/A ratio were significantly higher in patients with T2DM and HFpEF compared with the control group, while the LVEF was >50% in both groups (Figure 1c–e). Taken together, these observations suggest that patients with T2DM and comorbid HFpEF exhibit significant changes in fluid marker levels and cardiac structural-diastolic dysfunction. In animal studies, after eight weeks of sustained diabetes, evidence of structural and diastolic dysfunction was detected (Figures 3 and 7 and Table 3). However, systolic function remained unaffected, indicating that DCM was still in the compensatory stage [26,27]. Other studies have described that prolonged high glucose-induced myocardial infiltration, such as 12 weeks or longer, accompanied by STZ administration, leads to more severe systolic dysfunction [28]. The intricate pathophysiological mechanism underlying T2DM with HFpEF has not been elucidated, and early treatment options for T2DM with HFpEF are lacking. Herein, the elevated activity and level of FAP may play a critical role in the development of T2DM with HFpEF (Figure 1a and b). Besides, FAP activity demonstrated a stronger correlation and diagnostic value for T2DM with HFpEF than FAP level (OR 2.906 vs. 2.776, AUC 0.7641 vs. 0.7433) (Figure 1h and i and Table 2), consistent with the findings of previous studies [11,29]. Overall, these results indicate that FAP activity could serve as a potential novel biomarker for the diagnosis of T2DM with HFpEF. However, given that research on the relationship between FAP activity and T2DM-induced HFpEF is limited, further studies are warranted.

In line with both our observations and previously published studies [3,30], prolonged hyperglycemia accelerates and aggravates functional and structural cardiac alterations during the progression of diabetes. Of note, diabetes is associated with chronic low-grade inflammation and oxidative stress, leading to NF-κB activation, followed by the production of cytokines [3]. The present study examined the effects of pathological and physiological manifestations of T2DM-induced HFpEF, such as myocardial hypertrophy, myocardial fibrosis, inflammation, oxidative stress, apoptosis, and energy metabolism cardiac function in T2DM-induced HFpEF mice. As anticipated, FAP KO and inhibitor Talabostat attenuated these detrimental changes (Figures 3–7).

Beyond the heart, FAP is selectively expressed in activated stromal fibroblasts within pathological microenvironments. It is the most highly up-regulated in cancer-associated fibroblasts (CAFs) across multiple carcinomas, such as pancreatic, breast, and colorectal [31], than other fibrotic tissues such as liver cirrhosis, pulmonary fibrosis, and during wound healing [11]. In the healthy heart, FAP expression is at a basal level in resting state fibroblasts while completely unexpressed in cardiomyocytes and ordinary resident immune cells. But it becomes markedly induced in injury-activated cardiac fibroblasts and myofibroblasts [12]. In the bargain, FAP plays a vital role in mediating glucose and lipid metabolism. Its activity was significantly higher in the plasma of individuals with diabetes and obesity compared with healthy individuals. HFpEF is a metabolic disease, with comorbid conditions such as hyperlipidemia, hypertension [12], and hyperglycemia [4] contributing to myocardial dysfunction and the eventual development of HFpEF. Noteworthily, FAP KO did not affect the weight, blood glucose level, IPGTT, and ITT of T2DM-induced HFpEF mice, indicating that its cardioprotective effects occur independently of glycemic control. This conclusion is in agreement with that of previous research (Figure 2b, c, d and e) [32]. However, after treatment with FAP inhibitor Talabostat, T2DM-induced HFpEF mice showed weight loss and a nearly normal decrease in FBG levels, with significant differences in IPGTT and ITT compared with T2DM-induced HFpEF mice (Figure 7b, c, d and e). The peak and changes in blood glucose were well controlled. The distinct outcomes of FAP KO and the FAP inhibitor talabostat suggest significant differences between endogenous FAP KO and exogenous FAP inhibition. At the same time, this intriguing dissociation of FAP KO suggests that cardioprotective effects of FAP KO operate through glucose-independent mechanisms, likely involving the mitigation of T2DM-related damage in extracardiac organs. FAP exacerbates diabetic metabolic dysregulation by degrading GLP-1, thereby attenuating its protective effects on adipose tissue, liver, and skeletal muscle [33]. This process accelerates sustained glucotoxic damage to cardiomyocytes. Conversely, FAP KO or inhibition stabilizes GLP-1 signaling through reducing proteolysis to disrupt maladaptive inter-organ cross-talk. These mechanisms reveal FAP may be a novel therapeutic target for diabetic cardiomyopathy via influencing extracardiac target organs.

Under conditions of sustained hyperglycemia, dysregulated calcium homeostasis in cardiomyocytes leads to abnormal activation and pathological alterations of calcium-regulating enzymes, subsequently resulting in significant modifications of associated signaling pathways and downstream pathophysiological manifestations [9,34]. Previous studies have documented that diastolic dysfunction in HFpEF is related to impaired myocardial Ca^2+^ processing, which is manifested by the abnormal activation of Ca regulatory enzymes such as CaMK. Therefore, up-regulation of CaMKIIδ has been detected in animal models of DCM and in patients with diabetes [22]. Notably, while the CaMKIIδ-Calcineurin A-NFATc2 axis has been established as a critical regulator of fibroblast activation and proliferation [35,36], the direct interaction between this pathway and FAP expression remains unexplored. To our knowledge, no studies have yet systematically investigated whether FAP, as a canonical marker of activated fibroblasts, is functionally modulated by CaMKIIδ-Calcineurin A-NFATc2 signaling or conversely influences this pathway through feedback mechanisms. Our study is the first to explore the possible role between FAP and this pathway and to find that the abnormal increase of FAP activates the CaMKIIδ-Calcineurin A-NFATc2 signaling pathway independent of glycemic control (Figure 6a and b), thereby leading to the pathophysiological manifestations of T2DM-induced HFpEF and diastolic dysfunction [37–39]. After FAP KO, this signaling pathway was inhibited, resulting in the improvement in these changes in T2DM-induced HFpEF.

Overall, these findings highlight the potential of FAP activity inhibition in the treatment of DCM. Based on existing FAP vaccines [12] and the FAP inhibitor (Talabostat) used in preliminary clinical trials, treatments targeting FAP, especially those that inhibit its activity, are expected to offer clinical benefits to patients with T2DM-associated HFpEF. Simultaneously, FAP may serve as potential clinical biomarkers and new treatment for T2DM-associated HFpEF. Nevertheless, further studies are required to further promote the translational application of FAP in clinical practice.

## Conclusion

This study identified a new mechanism by which abnormal FAP activity and level promote diastolic dysfunction in T2DM-associated HFpEF. FAP KO concomitantly inhibited the CaMKIIδ-Calcineurin A-NFATc2 signaling pathway, thereby suppressing cardiac hypertrophy, fibrosis, inflammation, oxidative stress, apoptosis, and energy metabolism dysfunction, ultimately alleviating T2DM-induced HFpEF. Conclusively, FAP demonstrates dual clinical significance as both a novel biomarker for HFpEF screening in T2DM populations and a druggable target for developing precision therapies against diabetes-related cardiac dysfunction.

**Clinical Perspectives**

- The pathogenesis of heart failure with preserved ejection fraction (HFpEF) secondary to type 2 diabetes mellitus (T2DM) is complex, and the role of fibroblast activation protein (FAP) remains elusive.
- In the present study, FAP knockout (KO) attenuated the calmodulin-dependent protein kinase δ-Calcineurin A-NFATc2 signaling pathway and attenuated cardiac hypertrophy, fibrosis, inflammation, oxidative stress, apoptosis, and energy metabolism dysfunction, ultimately attenuating T2DM-induced HFpEF. Similarly, FAP inhibitor Talabostat reversed cardiac hypertrophy, remodeling, and oxidative stress, thereby improving T2DM-induced HFpEF.
- FAP may serve not only as a potential therapeutic target but also as a novel biomarker for early diagnosis and disease monitoring of T2DM-associated HFpEF. Additionally, its regulatory role suggests that FAP-based interventions (e.g., pharmacological inhibitors or vaccines) could represent promising strategies for preventing or mitigating the progression of HFpEF in diabetic patients.

## Supplementary material

Online supplementary table 1

Online supplementary table 2

Online supplementary figure 1

## Data Availability

All data and material used to support the findings of this study are available from the corresponding authors upon request.

## Abbreviations

CaMKIIδ: calmodulin-dependent protein kinase δ
FAP: fibroblast activation protein
FBG: fasting blood glucose
HF: heart failure
HFpEF: heart failure with preserved ejection fraction
LV: left ventricle
LVDS: left ventricular end-systolic diameter
LVEF: left ventricular ejection fraction
T2DM: type 2 diabetes mellitus
