# Supplementary material for: FAP deficiency attenuates T2DM-associated HFpEF by suppressing the CaMKIIδ-Calcineurin A-NFATc2 signaling pathway
Source: Clin Sci (Lond). 2025 Sep 2;139(17):923–40. doi: 10.1042/CS20256808 (PMC12599232; doi:10.1042/CS20256808)
Supplement: Online supplementary table 1 [file cs-139-17-CS20256808-s001.docx]

**Supplementary Table 1: A list of primary antibodies used in immunoblot analysis**

| **Antibody** | **Species** | **Dilution** | **Company** |
| --- | --- | --- | --- |
| TGF-β1 | human, mouse, rat | 1:2000 | Arigobio (ARG10002) |
| FAP | human, mouse, rat | 1:1000 | Abcam (ab53066) |
| p-mTOR | human, mouse, rat | 1:1000 | Cell Signaling Technology (#5536) |
| mTOR | human, mouse, rat | 1:1000 | Cell Signaling Technology (#2983) |
| p-ERK1/2 | human, mouse, rat | 1:1000 | Cell Signaling Technology (#4370) |
| ERK1/2 | human, mouse, rat | 1:1000 | Cell Signaling Technology (#4695) |
| NOX1 | human, mouse, rat | 1:1000 | Proteintech (17772-1-AP) |
| NOX2 | human, mouse, rat | 1:1000 | Proteintech (19013-1-AP) |
| NOX4 | human, mouse, rat | 1:1000 | Proteintech (14347-1-AP) |
| p-Smad2/3 | human, mouse, rat | 1:1000 | Cell Signaling Technology (#8828) |
| Smad2/3 | human, mouse, rat | 1:1000 | Cell Signaling Technology (#5678) |
| p-AMPKα | human, mouse, rat | 1:1000 | Cell Signaling Technology (#2535) |
| AMPKα | human, mouse, rat | 1:1000 | Cell Signaling Technology (#2532) |
| p-P65 | human, mouse, rat | 1:1000 | Wanleibio (WL02169) |
| P65 | human, mouse, rat | 1:1000 | Wanleibio (WL01980) |
| BNP | human, mouse, rat | 1:1000 | Abclonal (A2179) |
| CaMKIIδ | human, mouse, rat | 1:1000 | Abclonal (A9196) |
| p-CaMKIIδ | human, mouse, rat | 1:1000 | Abcam (ab182647) |
| Calcineurin A | human, mouse, rat | 1:1000 | Cell Signaling Technology (#2614) |
| NFATc2 | human, mouse, rat | 1:1000 | Abclonal (A14189) |
| Caspase 3 | human, mouse, rat | 1:1000 | Abclonal (A0214) |
| Bcl-2 | human, mouse, rat | 1:1000 | Abclonal (A19693) |
| Bax | human, mouse, rat | 1:1000 | Abclonal (A0207) |
| GAPDH | human, mouse, rat | 1:3000 | Cell Signaling Technology (#2118) |
