# Supplementary material for: FAP deficiency attenuates T2DM-associated HFpEF by suppressing the CaMKIIδ-Calcineurin A-NFATc2 signaling pathway
Source: Clin Sci (Lond). 2025 Sep 2;139(17):923–40. doi: 10.1042/CS20256808 (PMC12599232; doi:10.1042/CS20256808)
Supplement: Online supplementary table 2 [file cs-139-17-CS20256808-s002.docx]

**Supplementary Table 2: A list of PCR primer sequence used in Real-time quantitative PCR**

| Gene | species | Forward primer | Reverse primer |
| --- | --- | --- | --- |
| Collagen I | mouse | GAGTACTGGATCGACCCTAACCA | GACGGCTGAGTAGGGAACACA |
| Collagen III | mouse | TCCCCTGGAATCTGTGAATC | TGAGTCGAATTGGGGAGAAT |
| NOX1 | mouse | GCATCTGCTCTGTGCTTGAAT | GCTTATGGAAGGTGAGGTTGTG |
| NOX2 | mouse | TCAGTGAGCTTTCCCTGTGTC | GCATTTGCCTTCGGTGATGT |
| NOX4 | mouse | GCTTTGAACAGGATACATTCACACA | AGACCCATGCCCCAAATGAG |
| IL-1β | mouse | CTTCCCCAGGGCATGTTAAG | ACCCTGAGCGACCTGTCTTG |
| IL-6 | mouse | TTCCATCCAGTTGCCTTCTTG | TTGGGAGTGGTATCCTCTGTGA |
| TNF-α | mouse | ACCCTCACACTCACAAACCA | ACCCTGAGCCATAATCCCCT |
| BNP | mouse | GAAGGTGCTGTCCCAGATGA | CCAGCAGCTGCATCTTGAAT |
| ANF | mouse | CACAGATCTGATGGATTTCAAGA | CCTCATCTTCTACCGGCATC |
| GAPDH | mouse | TGTGTCCGTCGTGGATCTGA | TTGCTGTTGAAGTCGCAGGAG |
