# Supplementary material for: FAP deficiency attenuates T2DM-associated HFpEF by suppressing the CaMKIIδ-Calcineurin A-NFATc2 signaling pathway
Source: Clin Sci (Lond). 2025 Sep 2;139(17):923–40. doi: 10.1042/CS20256808 (PMC12599232; doi:10.1042/CS20256808)

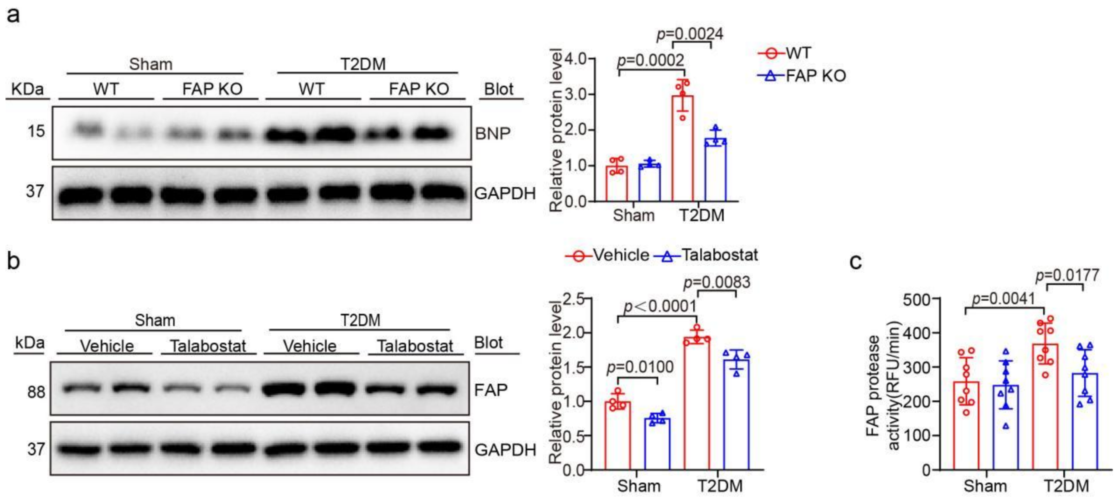


Figure S1

Figure S1. (a) Immunoblots of BNP in heart tissues from each group (left) and quantification of the relative protein (right, n = 4 per group). Data are expressed as mean±SD, and n represents the number of samples; (b) FAP protein level in heart of mice from continuing high fat diet for 8 weeks after T2DM induction by STZ intraperitoneal injection with WT or Talabostat mice in Sham, T2DM group (left) and quantification of the relative protein levels (right, n = 4 per group); (c) FAP activity in heart of mice from high fat diet for 8 weeks after T2DM induction by STZ intraperitoneal injection with WT or Talabostat mice in Sham, T2DM group (n = 8 per group). Data are expressed as mean±SD, and n represents the number of samples.

Fig 2 western blotting original figure

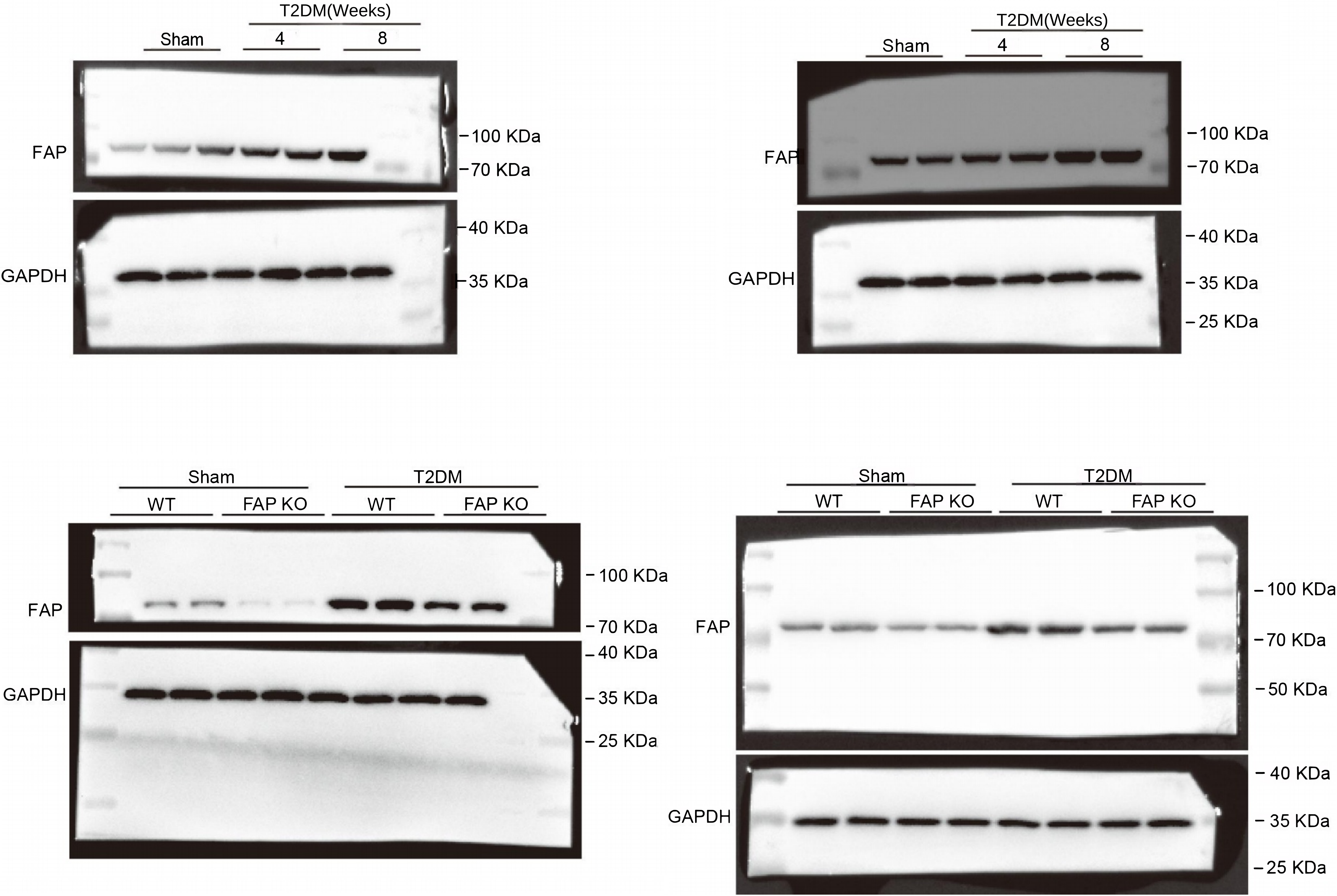

# Fig 4 western blotting original figure

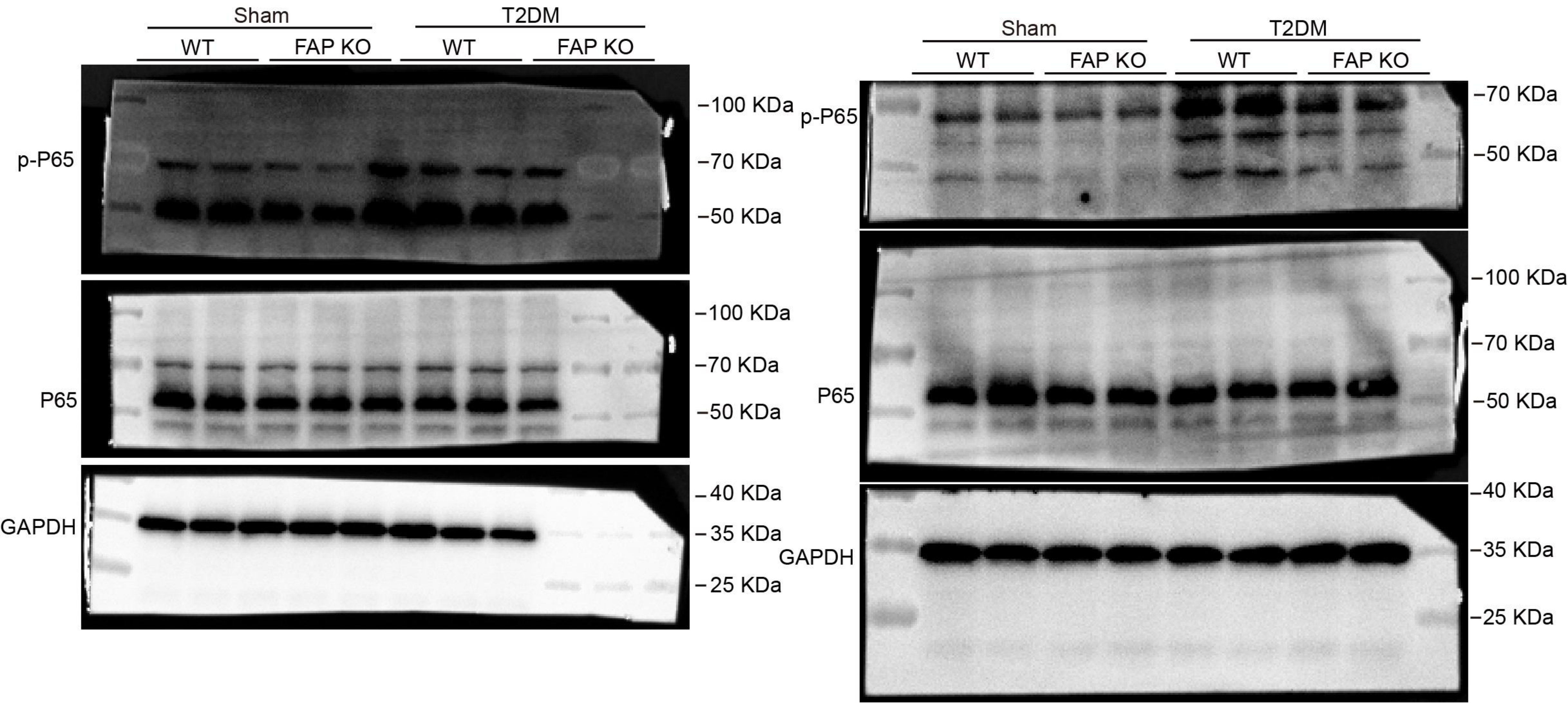

Fig 5 western blotting original figure

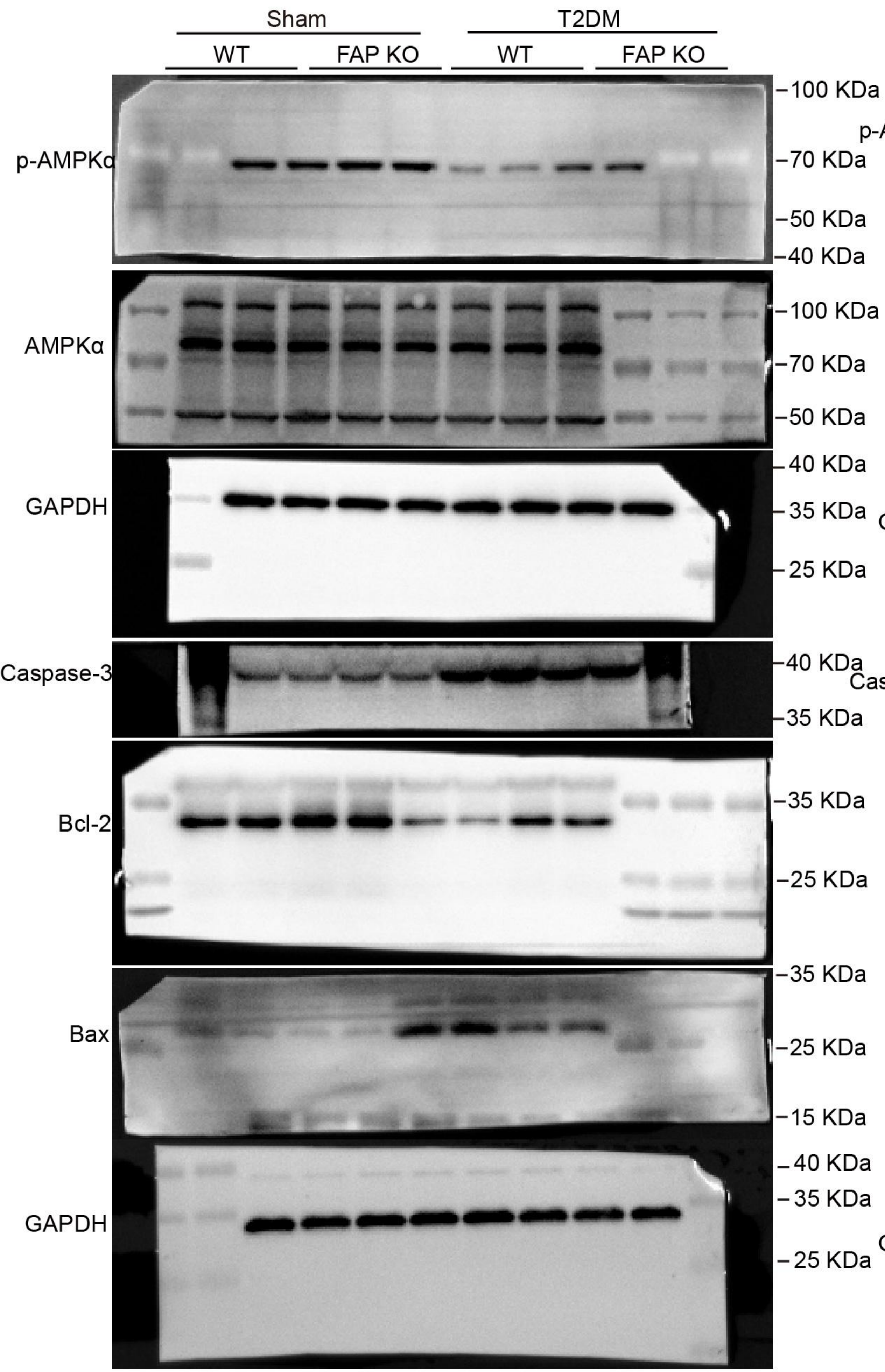


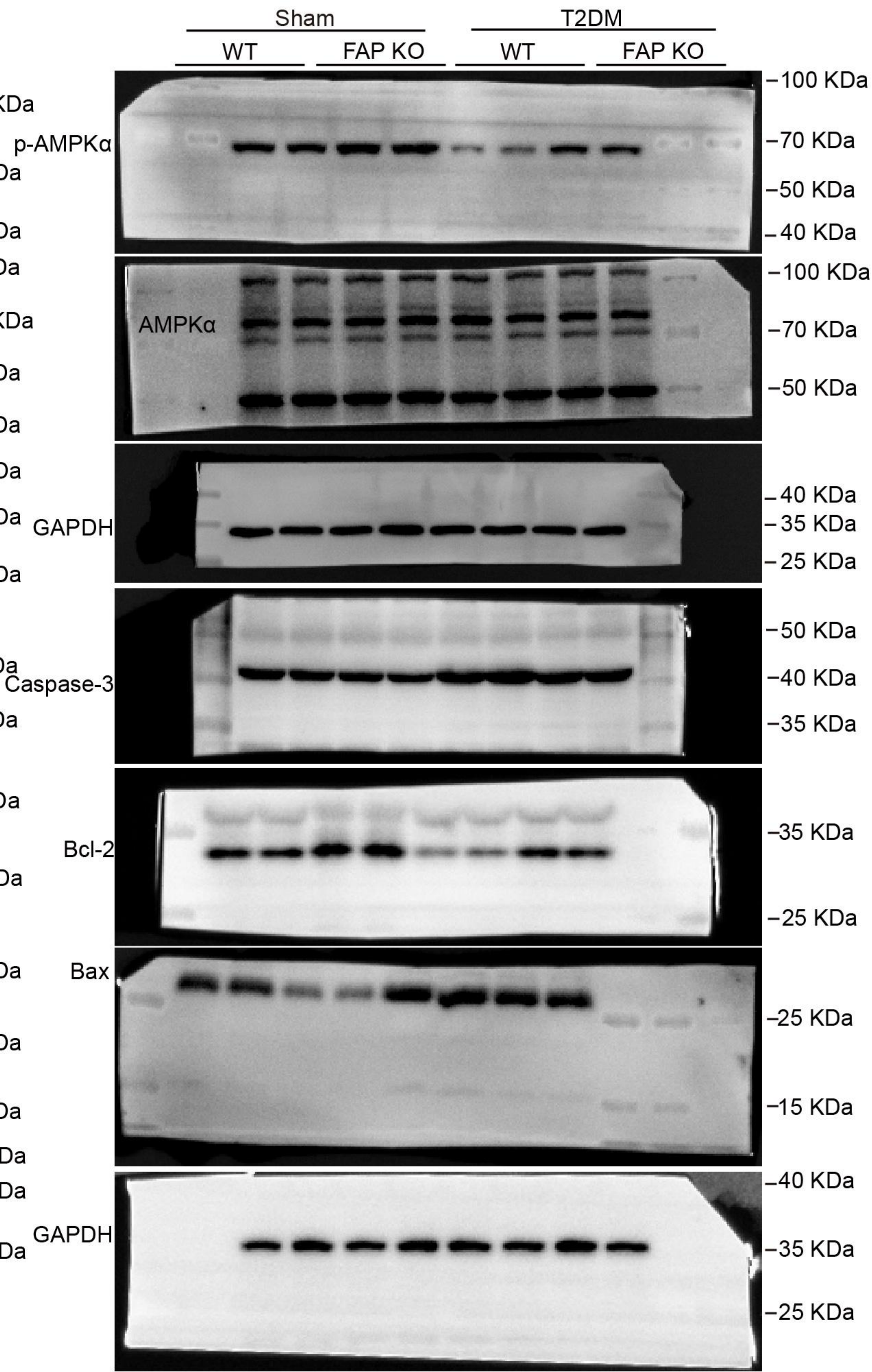

# Fig 6 western blotting original figure 1

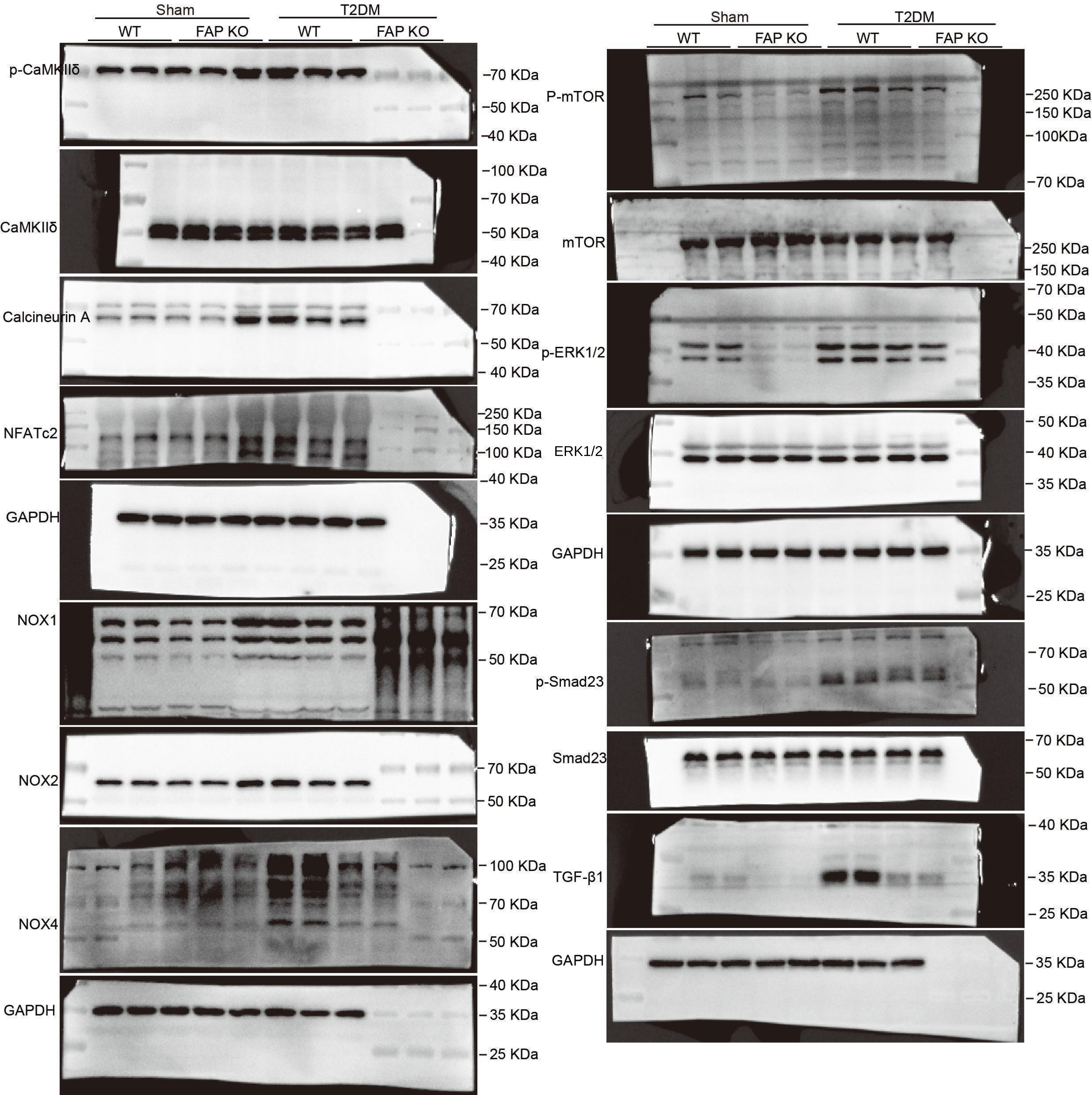

# Fig 6 western blotting original figure 2

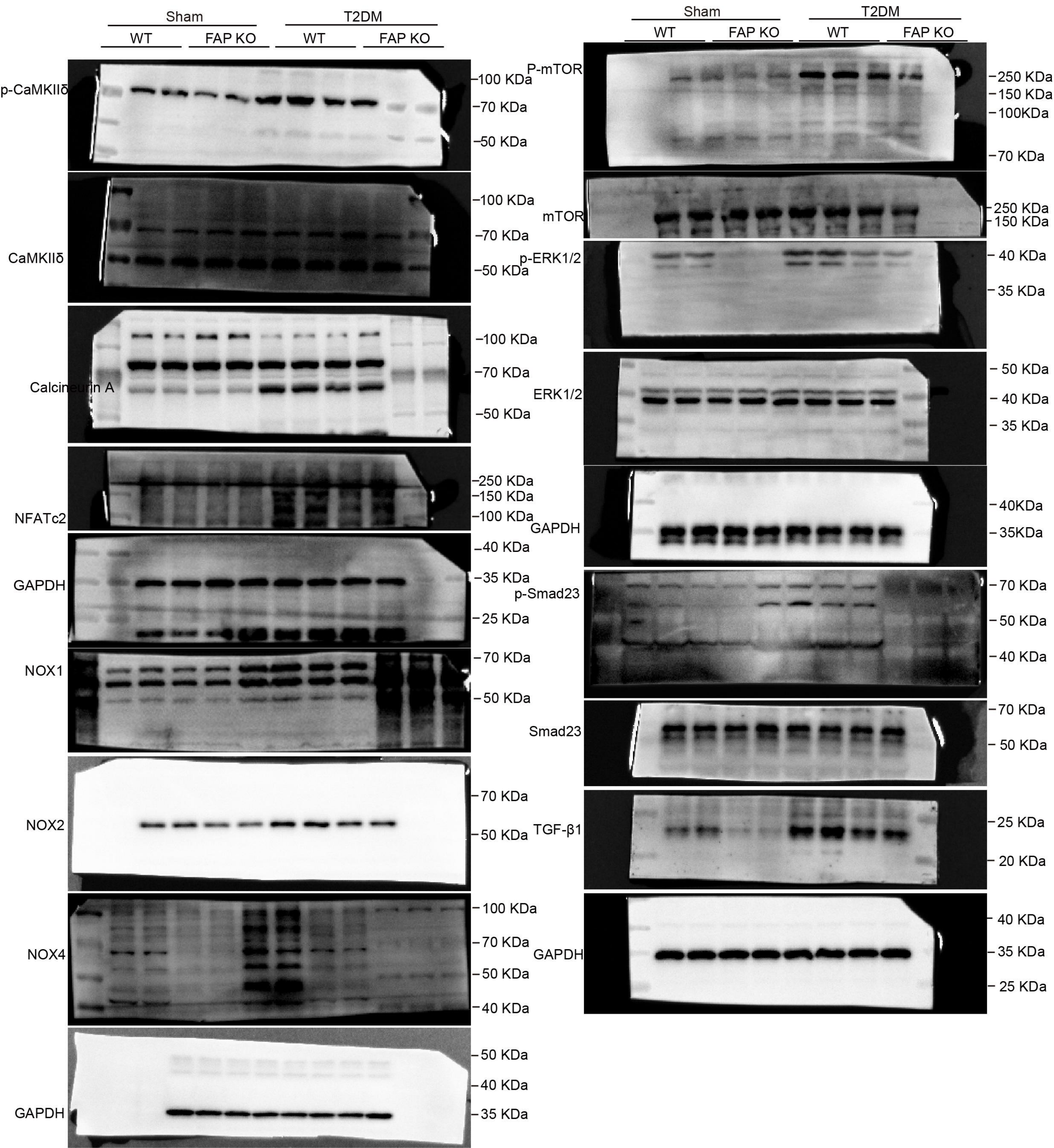

Fig S1 western blotting original figure

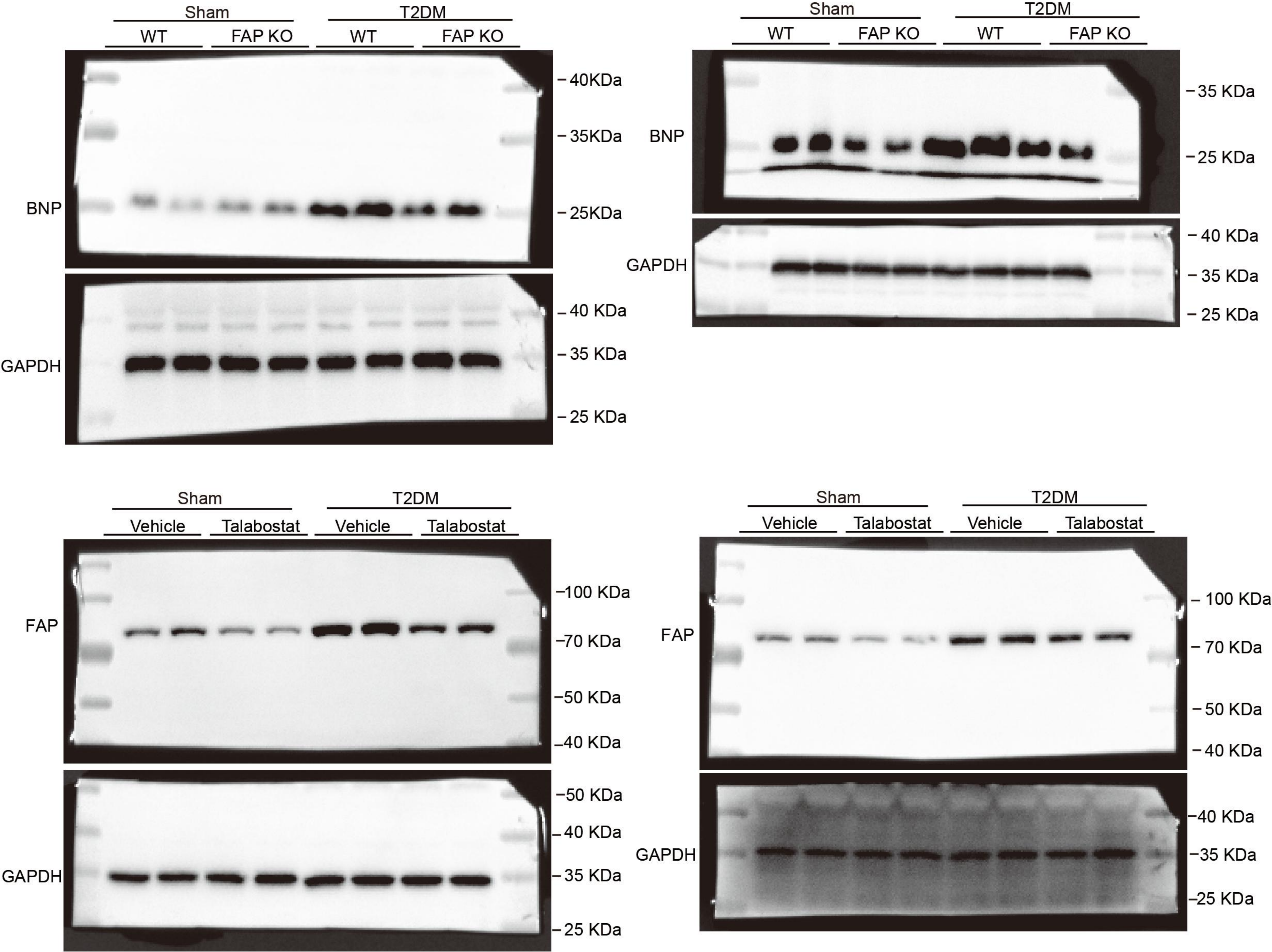

Supplement: Online supplementary figure 1 [file cs-139-17-CS20256808-s001.pdf]
